# Health risks to children from exposure to fecally-contaminated recreational water

**DOI:** 10.1371/journal.pone.0266749

**Published:** 2022-04-12

**Authors:** Timothy J. Wade, Benjamin F. Arnold, Ken Schiff, John M. Colford, Stephen B. Weisberg, John F. Griffith, Alfred P. Dufour

**Affiliations:** 1 U.S. Environmental Protection Agency, Office of Research and Development, Research Triangle Park, North Carolina, United States of America; 2 F.I. Proctor Foundation, University of California, San Francisco, San Francisco, California, United States of America; 3 Southern California Coastal Water Research Project, Costa Mesa, California, United States of America; 4 University of California, Berkeley, Division of Epidemiology, Berkeley, California, United States of America; 5 U.S. Environmental Protection Agency Office of Research and Development, Cincinnati, Ohio, United States of America; Purdue University, UNITED STATES

## Abstract

**Background:**

Children may be at higher risk for swimming-associated illness following exposure to fecally-contaminated recreational waters. We analyzed a pooled data set of over 80,000 beachgoers from 13 beach sites across the United States to compare risks associated with the fecal indicator bacteria *Enterococcus* spp. (measured by colony forming units, CFU and quantitative polymerase chain reaction cell equivalents, qPCR CE) for different age groups across different exposures, sites and health endpoints.

**Methods:**

Sites were categorized according to the predominant type of fecal contamination (human or non-human). Swimming exposures of varying intensity were considered according to degree of contact and time spent in the water. Health endpoints included gastrointestinal and respiratory symptoms and skin rashes. Logistic regression models were used to analyze the risk of illness as a function of fecal contamination in water as measured by *Enterococcus* spp. among the exposed groups. Non-swimmers (those who did not enter the water) were excluded from the models to reduce bias and facilitate comparison across groups.

**Results:**

Gastrointestinal symptoms were the most sensitive health endpoint and strongest associations were observed with Enterococcus qPCR CE at sites impacted by human fecal contamination. Under several exposure scenarios, associations between illness and *Enterococcus* spp. levels were significantly higher among children compared to adolescents and adults. Respiratory symptoms were also associated with *Enterococcus* spp. exposures among young children at sites affected by human fecal sources, although small sample sizes resulted in imprecise estimates for these associations.

**Conclusion:**

Under many exposure scenarios, children were at higher risk of illness associated with exposure to fecal contamination as measured by the indicator bacteria *Enterococcus* spp. The source of fecal contamination and the intensity of swimming exposure were also important factors affecting the association between *Enterococcus* spp. and swimming-associated illness.

## Background

Exposure to fecally-contaminated recreational waters can cause gastrointestinal, respiratory, and skin infections, and children may be particularly vulnerable [1–4]. Studies have shown that children stay in the water for a longer time and ingest more water during swimming than adults, resulting in an increased intensity and duration of exposure [5, 6]. In addition to being more highly exposed, children may be both more susceptible to contracting swimming-associated waterborne infections and more vulnerable to severe illness following infection. Children may lack prior exposures and pre-existing acquired immunity to waterborne pathogens making them more likely to become infected and experience symptoms following exposure. Some waterborne infections, such as Shiga-toxin producing *Escherichia coli* (e.g., *E*. *coli* 0157:H7), while relatively mild in most adults, can cause severe and sometimes life-threatening symptoms and complications in children, such as kidney failure (hemolytic uremic syndrome). Outbreaks of *E*. *coli* 0157:H7, *Cryptosporidium* spp., enteroviruses and other waterborne infections in both swimming pools and freshwater lakes disproportionally affect children [7, 8].

Untreated recreational waters (e.g., lakes and ocean beaches) are monitored and tested using fecal indicator bacteria including *Enterococcus spp*. (referred to hereafter as *Enterococcus*) and *Escherichia coli* (*E*. *coli*) [9]. These bacteria are not usually harmful in the recreational water environment, but because they are found in high concentrations in feces, they are used to indicate the potential presence of waterborne pathogens associated with sewage and other types of fecal contamination [10]. Epidemiology studies have demonstrated associations between the levels of *Enterococcus*, *E*. *coli*, and other fecal indicator organisms, in recreational waters and the risk of illness among swimmers (most commonly acute gastrointestinal symptoms) [11, 12]. Historically, these indicators have been measured using culturable methods, but in recent years, faster molecular methods based on quantitative polymerase chain reaction (qPCR) to measure these and other indicators have also been developed and applied in recreational water monitoring [13, 14].

Early epidemiology studies suggested children may be at a higher risk of illness following swimming exposure, but did not demonstrate a clear association with fecal indicator bacteria or other water quality indicators [1, 15]. Wade et al. provided epidemiological evidence that children had a higher risk of gastrointestinal illnesses associated with exposures to the fecal indicator bacteria *Enterococcus* (measured by qPCR cell equivalents, or qPCR CE) at four freshwater Great Lakes beaches [16]. Arnold et al. conducted a pooled analysis of epidemiological data collected by the U.S. EPA, University of California, Berkeley (UC Berkeley) and the Southern California Coastal Water Research Project (SCCWRP) from 13 sites (including both marine and freshwater) and including over 80,000 individuals [17]. They focused primarily on categorical exposure to *Enterococcus* using exposures at quartiles and at geometric means of 35 colony forming units (CFU) and 470 cell equivalents (CE) for culture and qPCR, respectively. They concluded that the associations between gastrointestinal illnesses and *Enterococcus* exposures were higher among children 10 years of age and under across the range of conditions studied.

These previous studies considered a somewhat limited range of exposure definitions (e.g., head immersion swimming, body immersion swimming) and one or two specific health endpoints. In this study, we utilized the pooled dataset from studies conducted by the U.S. EPA and UC Berkeley to further describe health risks among children in relation to recreational water exposures. Utilizing the 13-beaches dataset assembled by Arnold et al., we considered a broader range of exposures, health endpoints, and age categorizations than previous studies. We focused on log-linear exposure response relationships between levels of fecal contamination measured by *Enterococcus* (qPCR and culture) and illness to compare associations across age groups, beach types, exposure intensities and health endpoints.

## Methods

### Data sources

This assessment utilizes data collected from the National Epidemiologic and Environmental Assessment of Recreational Water Studies (NEEAR studies, 2003–2009)[16, 18, 19] and the UC Berkeley/SCCWRP epidemiology studies conducted between 2005 and 2010 [20–22]. The studies were conducted in the United States and Puerto Rico and sites included both freshwater (Great Lakes) and coastal marine beaches. The studies were designed to ensure comparability and integration and the datasets were previously combined and synthesized [17].

### Study design

The general epidemiological study design has been previously described in detail [16, 19]. In brief, individuals attending the beach were offered an opportunity to participate. Enrollment was open to households with at least one adult member present and adult individuals. Those who agreed to enroll provided written or oral informed consent. At study sites led by EPA (West Beach, Huntington Beach, Washington Park Beach, Edgewater Beach, Fairhope Beach, Goddard State Park Beach, Surfside Beach and Boquerón Beach). Written consent requirements were waived by the Institutional Review Board and an adult household member consented to provide information about household members on behalf of the family, including children. At study sites led by UC Berkeley/SCCWRP (Mission Bay, Doheny Beach, Malibu Beach, and Avalon Beach), signed/written consent was obtained for all adult members present and adults gave consent for participation for children under 18 years of age. After enrollment, participants completed a baseline health and demographic survey, and were interviewed again upon leaving to ascertain information regarding swimming and other exposures during the visit. They were then interviewed again by phone approximately 10–14 days later and asked about any new illness symptoms they experienced since the beach visit. Symptoms included gastrointestinal (diarrhea, nausea, vomiting), respiratory (cough, cold) skin (rash, skin infection), ear and eye irritations. Water samples were collected at several times and locations during the day and tested for indicators of fecal contamination using both standard methods (e.g., *Enterococcus* CFU) and rapid molecular methods using qPCR. The study protocols were reviewed and approved by institutional review boards at the University of California, Berkeley, the University of North Carolina, Chapel Hill, and the Centers for Disease Control and Prevention and received ethical review and approval from the US Environmental Protection Agency’s human subjects research review office.

Participants were categorized according to swimming activity: those without any water contact (non-swimmer) and various degrees of water contact, ranging from wading, body immersion, head immersion, etc. Exposure was further classified according to levels of fecal contamination in the water on the day of the beach visit. Incidence of new illness symptoms over the follow up period was compared to water quality levels and swimming exposures.

### Data analysis

The analysis approach was similar to that described in previous studies [18, 19]. The association between water quality and illness symptoms was modeled using logistic regression, where the outcome was a binary indicator of illness (expressed in the logistic model as the odds or log-odds of illness), and the primary exposure was the measured water quality (log_10_ transformed to reduce skewness) on the day of exposure. Logistic regression models were restricted to exposed swimmers only, considering various definitions of “swimmer” described below. To provide consistency in comparisons across the various age groups and exposure scenarios, we assumed associations between *Enterococcus* and the log-odds of illness were linear on the log scale. Potential confounding factors were those identified in previous studies, and a final model was selected using backwards stepwise selection, minimizing Akaike’s Information Criterion [23]. Factors in the initial model for gastrointestinal illness included: beach site, contact with other ill people, chronic illness, consumption of undercooked meat, age, gender, recent precipitation, race, other swimming, and contact with unfamiliar animals. The initial set of factors was modified for models with respiratory illness and rash as the outcomes to include use of sunblock, insect repellant, and allergies. Because the sampling design focused on households, standard errors for the coefficients and effect estimates from the final regression models were adjusted for non-independence clustering by household using cluster-robust standard errors [24–26].

Associations between *Enterococcus* and illnesses among swimmers were presented as odds ratios (OR), which represent the increase in the risk (odds) of the illness per log_10_ increase in daily average of *Enterococcus* CFU or qPCR CE among the exposed group.

The strength (indicated by the magnitude of the odds ratios) and statistical significance of the associations were compared across age categories, varying the key parameters shown in Table 1. Interaction terms between the water quality measure and an indicator variable for age category were included to quantify the magnitude and significance of the differences in associations between water quality and the health endpoint by age category. Children (defined by age categories shown in Table 1) were compared to older participants (children and adults), and to adolescents and adults 18 years of age and older. The p-value associated with the multiplicative interaction term was used as a general guide to assess whether the effects were substantially different among children. Generally, two-sided p-values less than 0.05 were considered statistically significant, however, because of the observational nature of the study and the multiple analyses conducted, p-values and confidence intervals were not considered strict criteria of statistical significance.

**Table 1:** Alternate categorizations for key parameters.

Parameter	Definition
Illness outcome	▪ Gastrointestinal illness (NEEAR-GI)
	▪ Diarrhea
	▪ Vomiting
	▪ Severe gastrointestinal illness
	▪ Respiratory illness
	▪ Cold
	▪ Sore throat
	▪ Rash
Swimming exposure	▪ Any contact with water
	▪ Body immersion
	▪ Swallowing water
	▪ At least 30 minutes in water
	▪ At least 60 minutes in water
Water quality indicator	▪ Enterococcus qPCR-CE^a^ (geometric mean-daily average)
	▪ Enterococcus CFU^b^ (geometric mean- daily average)
Age category^c^	▪ All ages
	▪ 12 and under
	▪ 10 and under
	▪ 6 and under
	▪ 4 and under
	▪ 13 and over
	▪ 18 and over
Source/site	▪ All sites
	▪ Sites with likely human source
	▪ Sites with likely human source, excluding tropical site
	▪ All NEEAR sites
	▪ NEEAR point source sites
	▪ NEEAR core sites (excluding tropical and non-point source site)

^a^quantitative polymerase chain reaction cell equivalents.

^b^colony forming units^.^

^c^additional age groups considered, see text for description.

Data were analyzed and managed using R-Studio (version 1.4.1717) installed with R version 4.1.0 and Stata version 17.0. Plots were created in R-Studio using the *ggplot2* package [27].

### Age categories, health endpoints, swimming exposures, and study sites

We considered alternate swimming exposures and age categories, a wide range of health endpoints, and different groupings of study sites based on likely source of fecal contamination. Alternate categorizations for these key parameters are shown in Table 1.

We included several definitions of swimming beyond standard definitions such as body or head immersion in water including swallowing water and time spent in the water (greater than 30 minutes and greater than 60 minutes). The latter categories entail more intensive and prolonged exposures and may result in an increased potential for infection with any waterborne pathogens present.

Most previous analyses from these datasets focused primarily on a derived definition gastrointestinal illness (referred to as NEEAR-GI to distinguish it from other definitions of GI illness) defined as any of the following: diarrhea (three or more loose stools in a 24-hr period), vomiting, nausea and stomachache, and nausea or stomachache that affect regular activity (inability to perform regular daily activities) [18]. In this analysis, we also considered alternate illnesses including respiratory illness, alternative definitions of gastrointestinal illness and individual gastrointestinal and respiratory symptoms for a more comprehensive comparison of risks between children and other age groups. “Severe” gastrointestinal illness was defined as gastrointestinal symptoms that lasted three or more days or resulted in a visit to the hospital, doctor’s office or emergency room. Respiratory illness was defined as two or more of the following symptoms: cold, cough, sore throat, runny nose and fever.

Several age categories were defined to characterize children’s risk and to contrast these risks with older children (over 12) and adolescents and adults (18 and over). Most of these age groups were overlapping (e.g., 12 and under, 10 and under, etc.). This was done because the primary goal was to compare a range of alternate categorizations for children with the rest of the population and to older adolescents and adults (18 and over). For additional comparison, several alternate and narrower age categories for children (age groups: 4–12; 4–10; 6–10 and 6–12 years) were considered.

Recreational waters can be contaminated with fecal microorganisms from both human and non-human sources, and standard approaches to measure fecal indicator bacteria such as *Enterococcus* cannot distinguish between sources. Human sources of fecal contamination are generally considered to be the highest risk as they usually contain higher levels of disease causing microorganisms, such as human enteric viruses, and studies have shown that risk of adverse health effects can vary depending on the source of the fecal contamination [20, 28, 29]. In this analysis, suspected human impacted sites and scenarios were considered separately. We used the classification similar to that used by Benjamin-Chung et al. to categorize sites in this dataset as likely human impacted [30]. All NEEAR study beaches except Surfside Beach in South Carolina were considered human-impacted because of the presence of nearby wastewater treatment discharges. The wastewater treatment plants impacting the NEEAR study beaches have been described previously [16, 19]. Each site was impacted by point sources of discharge from one or more wastewater treatment plants, most of which provided secondary or tertiary treatment. Surfside Beach was studied specifically to represent a site without any known point sources of human fecal contamination, and primarily impacted by urban runoff [31]. At Doheny Beach, days when a sand berm was open and allowed the flow of the San Juan Creek into the surf zone were considered as human-impacted [32]. At Avalon Beach, wastewater from a faulty sanitary sewer system discharged into submarine groundwater through the sand and was moderated by tidal conditions [22]. Days when groundwater flow was above the median were considered human-impacted and those when it was below median flow as not human-impacted, consistent with previous studies at this beach [22, 30]. A subsequent study confirmed the presence of markers of human fecal contamination from water samples at both sites [33]. We classified all days at Malibu and Mission Bay beaches as not human-impacted because there were no known sources of human fecal discharges [20, 21, 34].

At Boquerón beach in Puerto Rico, the *Enterococcus* qPCR signal was often inhibited which affected the ability to interpret the results and to develop an association with swimming-associated illness [31]. In addition, despite being located near wastewater effluent, water samples collected near the beach also indicated that over the time period of the study, wastewater effluent was not likely impacting the beach directly [35]. Because of these issues, site categorizations with and without this site were considered.

Finally, the core NEEAR study beaches (four freshwater and three marine beaches studied between 2002 and 2007) were also studied separately as these were specifically selected for their consistent impacts from point sources of human fecal contamination with discharging populations of at least 15,000 individuals [16, 19]. Fecal contamination at these sites was expected to be less likely to be driven by sporadic events such as wet weather and specific beach conditions. The beach sites, study population, location and the categorizations are described in S1 and S2 Tables.

### Water quality measurements

Water sampling and testing methods have been described previously in detail [14, 16, 18, 20, 21, 32]. Water samples were collected at several times and locations each study day. Tests for *Enterococcus* were done by culture (quantified by CFU) using EPA Method 1600 and qPCR (quantified by CE) using EPA Methods 1611 and 1609. Exposure measures were summarized using the same approach described by Arnold et al [17]. In brief, *Enterococcus* estimates were log_10_ transformed and then averaged to create estimates of the water quality at a particular beach on a given day. This analysis used the mean of all log_10_ *Enterococcus* measures from a given day as the primary estimate of exposure of fecal contamination. Previous analyses have demonstrated the daily average water quality was an equivalent or better predictor of swimming-associated illness than time and location-specific averages [16, 18, 19].

Although water samples were tested for other indicators of fecal contamination (e.g., *E*. *coli*, *Bacteroides* spp., coliphage), this analysis was restricted to *Enterococcus* as it was the only indicator measured at every beach site.

## Results

### Study population

Across all beach sites, there were a total of 83,452 participants. Children 12 and under made up 20–25% of the study population across the various site categories. Seventy percent had at least some contact with water and 27% stayed in the water 60 minutes or more. Information on study populations, age groups, health endpoints and site classifications are shown in S3 Table.

### Water quality

Water quality samples were collected and tested for *Enterococcus* CFU on 360 study days across all sites and for *Enterococcus* qPCR CE on 347 days (no qPCR tests were conducted on 8 days at Doheny Beach and 5 days at Mission Bay). Daily geometric means of *Enterococcus* CFU ranged from below detection (assigned a value of 0.1 CFU per 100 ml) to 3,100 CFU per 100 ml. Daily geometric means of *Enterococcus* qPCR CE ranged from below detection (0.1 per 100 ml) to 7,315 per 100 ml. Water quality distributions for the various site categories are shown in S4 Table.

### Associations between water quality and swimming-associated illness

Results are presented in forest plots of odds ratios with 95% confidence bounds and with the null or no- effect value of 1 indicated with a dashed line. To improve the readability and clarity of the plots, some age and site categories were excluded from the plots shown in the main text. Notably strong and significant associations not shown in the plots were mentioned in the text or in the Supporting Information.

#### Gastrointestinal illness (NEEAR-GI)

**Fig 1:**
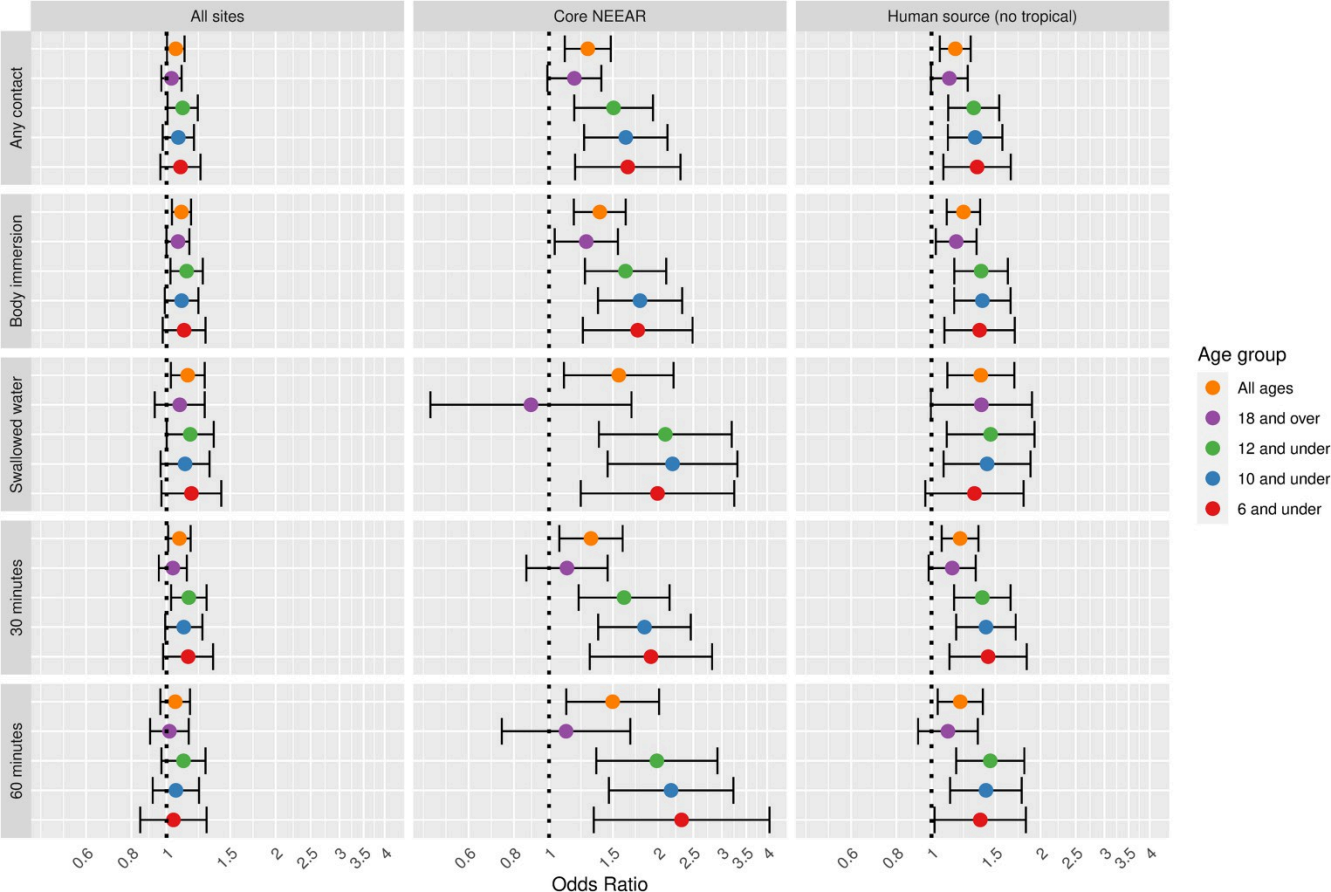
Odds ratios for NEEAR-GI illness among swimmers associated with a 1-log_10_ increase in *Enterococcus* qPCR CE.

**Fig 2:**
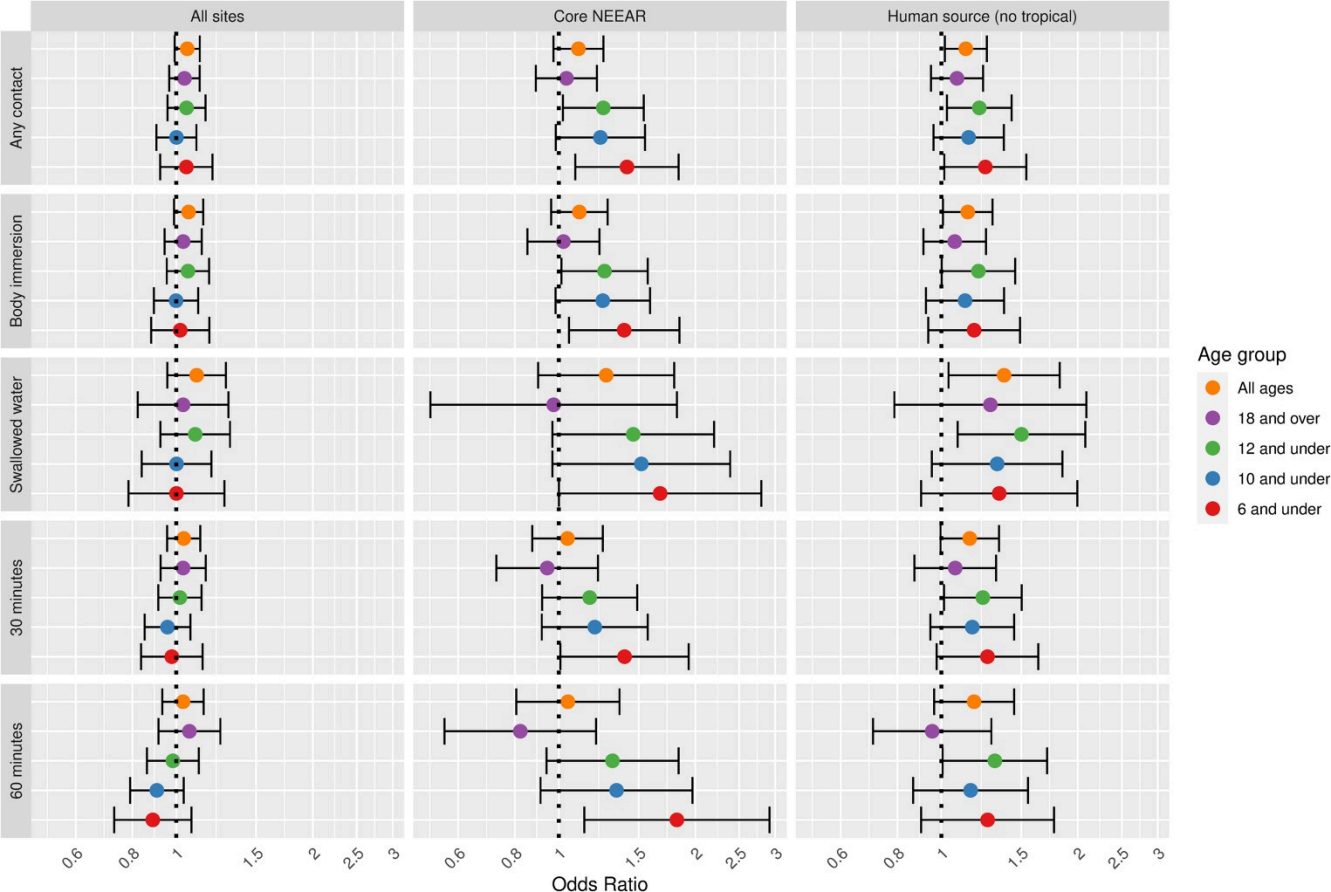
Odds ratios for NEEAR-GI illness among swimmers associated with a 1-log10 increase in *Enterococcus* CFU.

Figs 1 and 2 illustrate the associations between GI illness (NEEAR-GI) associated with a log_10_ increase *Enterococcus* qPCR CE and *Enterococcus* CFU, respectively for selected site categories (all sites, core NEEAR sites and human impacted sites), age groups and exposure categories.

The odds ratios for the association between NEEAR-GI and *Enterococcus* qPCR CE at all sites and among all ages for body immersion swimmers was 1.10 (95% CI 1.03–1.17). Odds ratios tended to be higher among children and with more intense swimming exposures (e.g., swallowing water or spending at least 60 minutes in the water). This trend was most pronounced in the core NEEAR sites and to a lesser extent at the likely human impacted sites. At the core NEEAR sites, for exposures such as swallowing water and spending over 60 minutes in the water, the overall risk was driven by the effect among children (Fig 1), as there was no association among those 18 and over. Among children six years of age and under who spent at least 60 minutes in the water, the odds ratio for NEEAR-GI associated with a log_10_ *Enterococcus* qPCR CE was 2.32 (95% CI 1.33–4.06) at the core NEEAR sites, whereas the odds ratio was (1.11, 95% CI 0.74–1.68) for those 18 and over. Similar associations were observed for children under 8 and children under 4 at the core NEEAR sites (S1 Fig). Significant interaction effects comparing children to those 18 and over were observed at the core NEEAR sites among those who swallowed water, for the age groups 12, 10, 8 and 6 and under; and among those who were in the water at least 60 minutes for the age groups 12 and under and 10 and under. When all sites were considered together, associations were attenuated, although still generally elevated, and differences across exposure categories and age groups were not as apparent.

For all sites and all ages, the association between GI illness and *Enterococcus* CFU were positive (Fig 2) but were not statistically significant. At likely human impacted sites odds ratios were increased, particularly among those who swallowed water (OR = 1.37, 95% CI 1.04–1.82). There was evidence for increasing risk with younger ages at the core NEEAR sites. For example, among children 6 and under (Fig 2) and children 4 and under (S2 Fig) spending 60 minutes or more in the water the odds ratios were 1.82 (95% CI = 1.14–2.91) and 2.06 (95% CI = 1.09–3.87), respectively, which differed significantly compared to the associations among those 18 and over (p = 0.05 and p = 0.04, respectively).

#### Diarrhea

**Fig 3:**
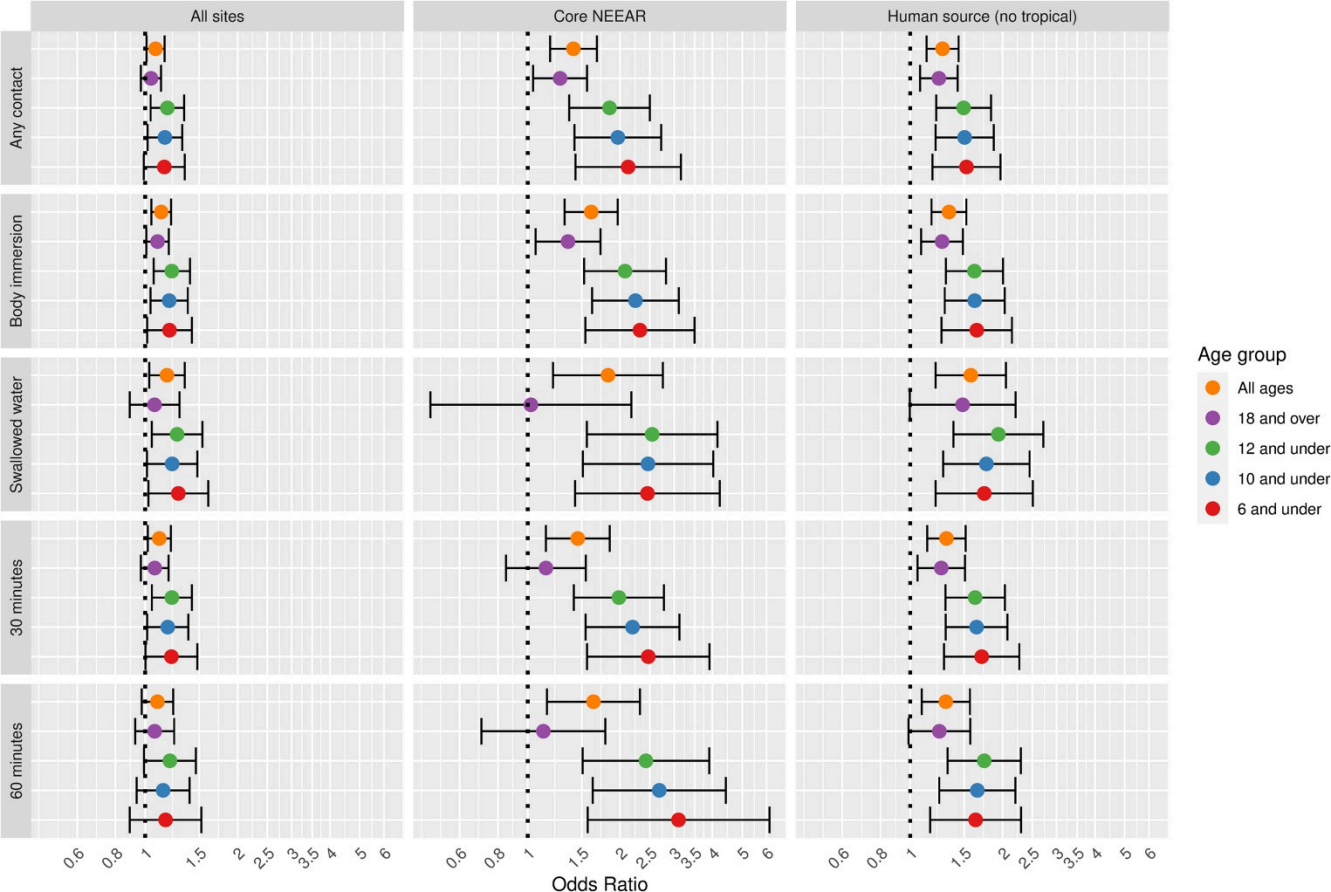
Odds ratios for diarrhea among swimmers associated with a 1-log10 increase in *Enterococcus* qPCR CE.

**Fig 4:**
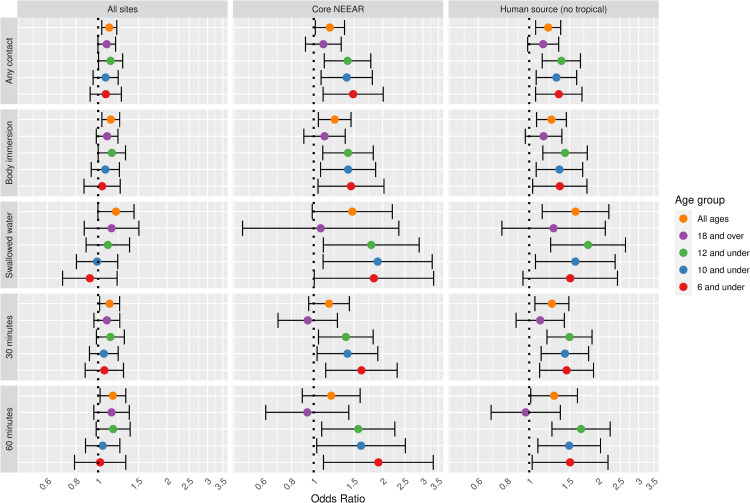
Odds ratios for diarrhea among swimmers associated with a 1-log10 increase in *Enterococcus* CFU.

Associations between diarrhea and *Enterococcus* qPCR CE showed trends similar to NEEAR-GI but odds ratios were generally higher (Fig 3). Across all sites, the odds ratio for the association between *Enterococcus* qPCR CE and diarrhea was 1.13 (96% CI 1.05–1.21). Odds ratios were higher at sites with likely human sources particularly for more intensive exposures such as swallowing water (OR = 1.58, 95% CI 1.21–2.05). The largest odds ratios were among children at the core NEEAR sites. For example, the strongest associations were among children 6 and under (Fig 3) and 4 and under (S3 Fig) who spent at least 60 minutes in the water the odds ratios were 3.10 (95% CI = 1.57–6.14) and 3.13 (95% CI = 1.41–6.96), respectively. Significant interaction effects were observed at the core NEEAR sites. Among those exposed more than 30 minutes, children in age groups 10, 8, 6 and 4 and under all had significantly higher risks compared to those 18 and over (Fig 3 and S3 Fig). Statistically significant interaction effects were also observed between children and those 18 and over among swimmers who swallowed water (age categories 6 and under and 12 and under) and had body immersion exposures (age categories 8 and 6 and under). For example, among children 12 and under who swallowed water, the odds ratio for diarrhea was 2.54 (95% CI 1.56–4.15) whereas among those 18 years and over the odds ratio was 1.02 (95% CI 0.48–2.18).

Diarrhea was associated with *Enterococcus* CFU at all sites and among all age groups (e.g., OR = 1.14, 95% CI = 1.04–1.24 among body immersion swimmers, Fig 4). At likely human impacted sites and core NEEAR sites there were higher odds ratios among younger age groups and more intense swimming exposures. Among those who swallowed water at likely human impacted sites the odds ratio for all subjects was 1.59 (95% CI = 1.14–2.23), and the highest odds ratios were among young children at the core NEEAR sites (OR = 1.92, 95% CI = 1.10–3.33).

#### Severe gastrointestinal illness

**Fig 5:**
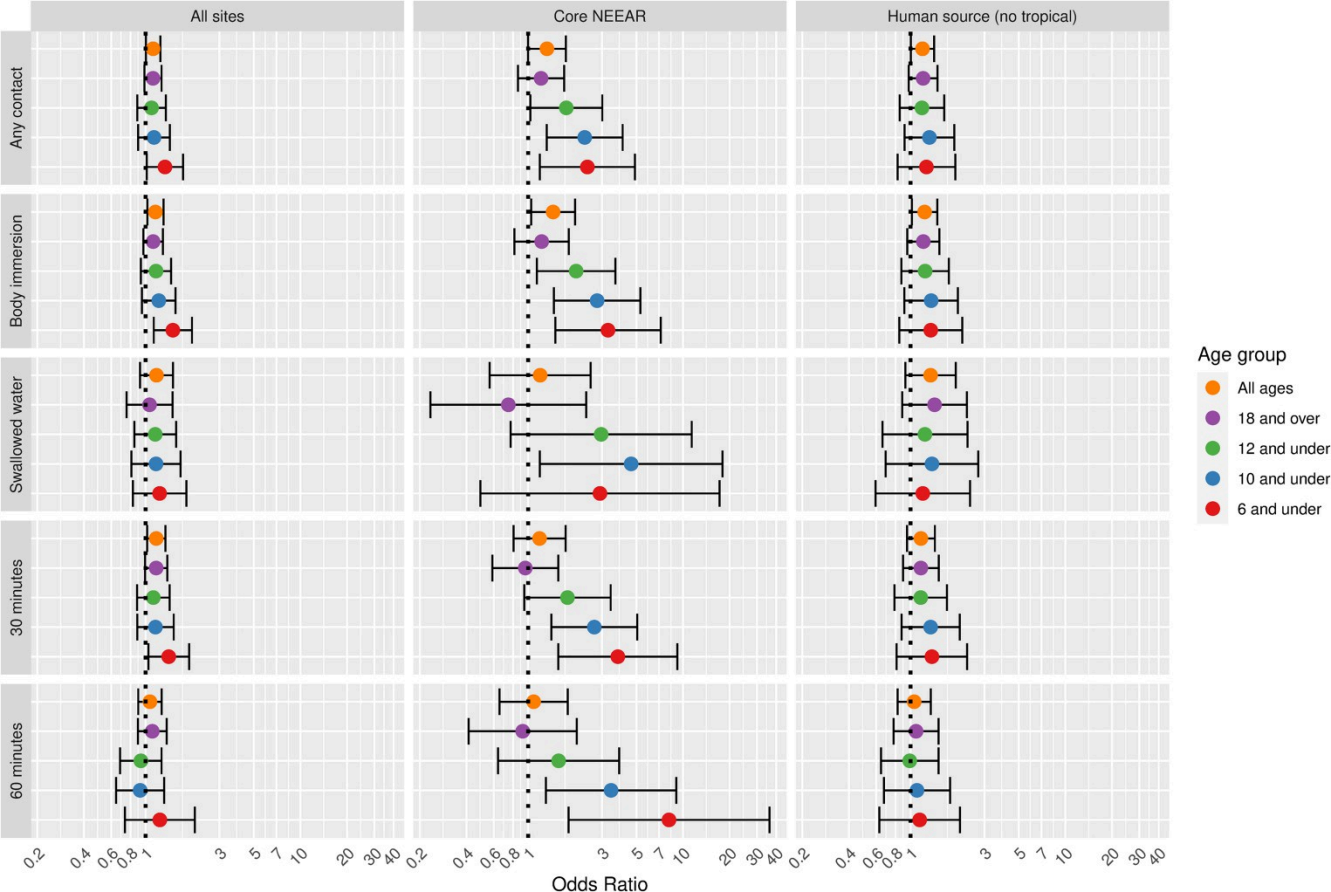
Odds ratios for severe gastrointestinal illness among swimmers associated with a 1-log10 increase in *Enterococcus* qPCR CE.

Severe GI illness, defined as a GI illness episode that lasted three or more days or resulted in a visit to the hospital, doctor’s office or emergency room, accounted for about 20% of all GI illness episodes (S3 Table). Because there were relatively fewer cases, estimates were imprecise with wide confidence bounds (Fig 5). At all sites and among all participants, associations between severe GI illness and *Enterococcus* qPCR CE among any contact and body immersion swimmers were statistically significant (OR = 1.12; 95% CI = 1.00–1.25; and OR = 1.16; 95% CI = 1.03–1.31) and was elevated further among body immersion swimming children 6 and under (OR = 1.50, 95% CI = 1.13–1.99). Increased odds ratios were observed again at the core NEEAR sites, among children and for more intensive exposures (Fig 5). For example, among children 6 and under who stayed in the water 60 minutes or longer the odds ratio of severe GI illness associated with *Enterococcus* qPCR CE was 8.13 (95% CI = 1.82–36.2), although due to few cases of illness for this category, the estimates are imprecise with wide confidence bounds.

Associations between *Enterococcus* CFU and severe GI illness were generally not statistically significant (see S4 Fig).

#### Other gastrointestinal symptoms

Associations between *Enterococcus* qPCR CE and vomiting, nausea and stomachache, are shown in S5–S7 Figs. Associations between *Enterococcus* qPCR CE and vomiting and nausea were generally not statistically significant, whereas the general patterns of the associations with stomachache more closely followed the associations observed for GI illness and diarrhea. Associations between vomiting, nausea, and stomachache with *Enterococcus* CFU were generally not significant (S8–S10 Figs).

#### Respiratory illness

**Fig 6:**
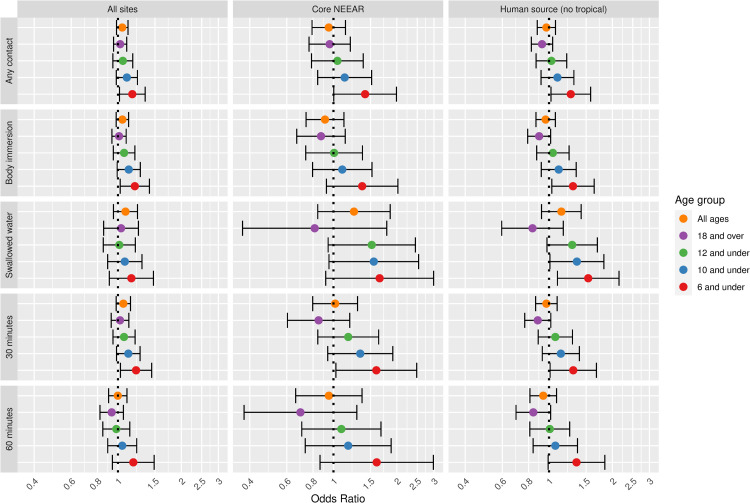
Odds ratios for respiratory illness among swimmers associated with a 1-log_10_ increase in *Enterococcus* qPCR CE.

**Fig 7:**
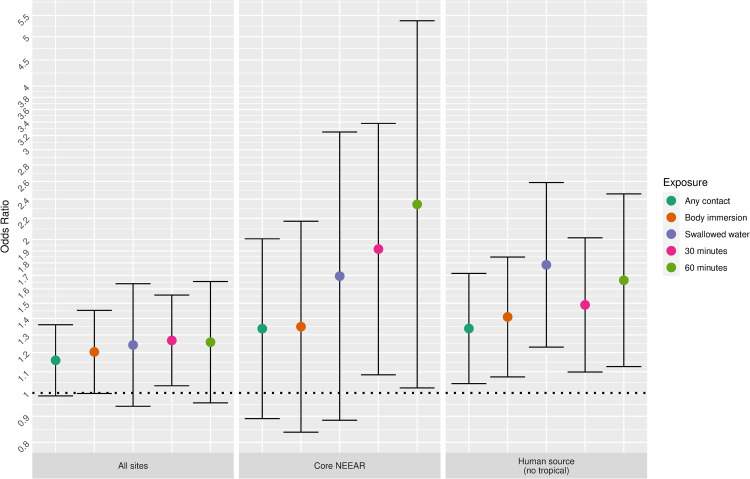
Odds ratios for respiratory illness among swimmers associated with a 1- log_10_ increase in *Enterococcus* qPCR CE among children 4 years and under.

Associations between upper respiratory illness and *Enterococcus* qPCR CE are shown in Fig 6. Most associations were not significant across different age groups, exposures and sites, although there was of evidence higher risks for younger children, particularly at likely human impacted sites and the core NEEAR sites. The odds ratio for respiratory illness among children 6 and under who swallowed water associated with *Enterococcus* qPCR CE exposure at likely human impacted sites was 1.54 (95% CI 1.10–2.15). Elevated odds ratios were also observed the youngest children, age 4 and under, at the core NEEAR sites and likely human impacted sites (Fig 7).

Associations for individual respiratory symptoms: sore throat, cough and cold are shown in S11–S17 Figs.

**Fig 8:**
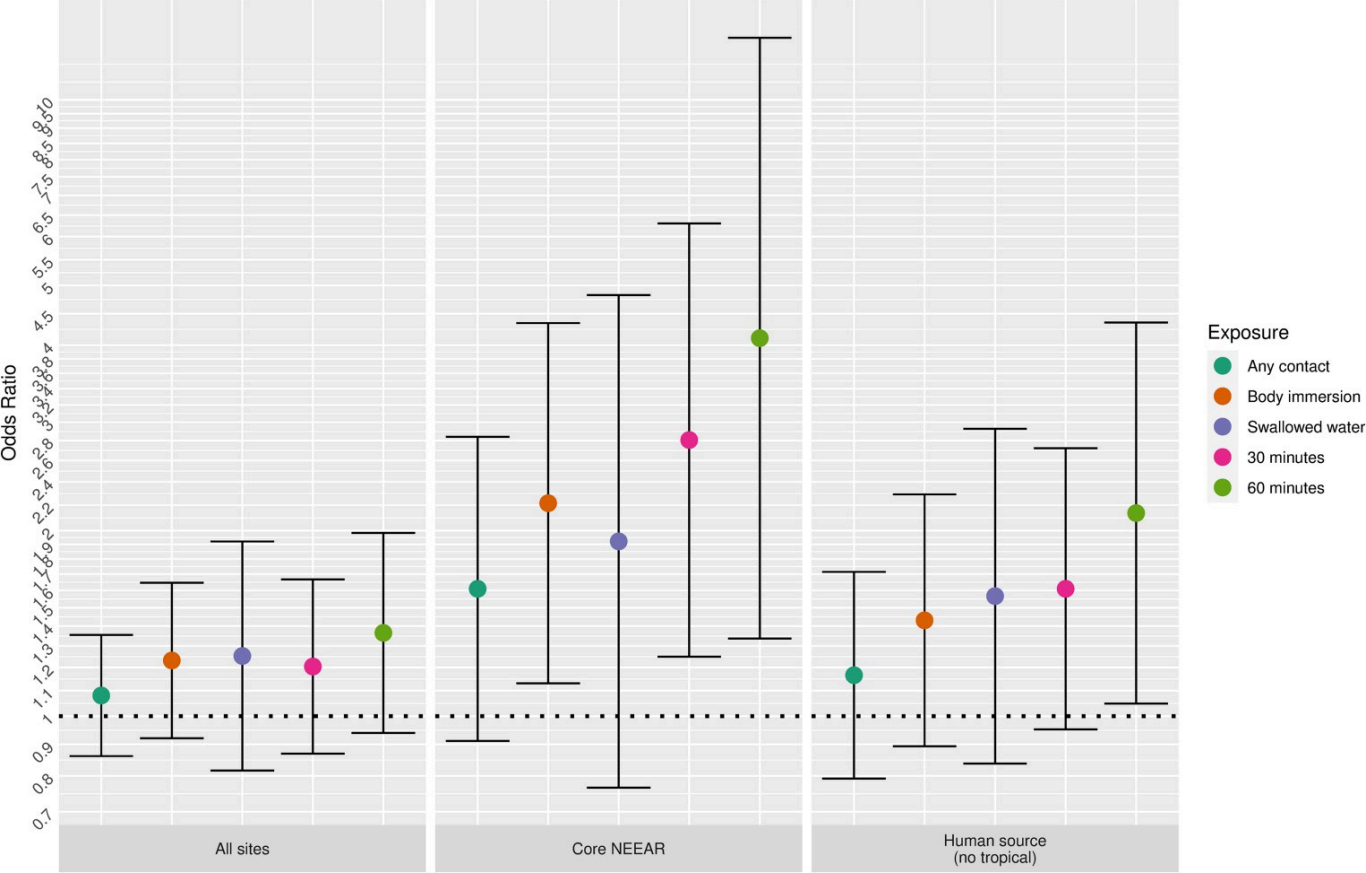
Odds ratios for cough among swimmers associated with a 1-log_10_ increase in *Enterococcus* CFU among children 4 years and under.

Most associations with respiratory illness and individual respiratory symptoms and *Enterococcus* CFU were not significant among all subjects, adults and older children (See S14–S17 Figs). Respiratory illness was associated with *Enterococcus* CFU among children 4 and under who spent 60 minutes or more in the water at the core NEEAR sites (OR = 2.93, 95% CI = 1.19–7.21)) and among children 12 and under at likely human impacted sites. Cough was also associated with *Enterococcus* CFU at the core NEEAR sites among young children 4 and under (Fig 8 and S17 Fig), although due to relatively few cases in these subsets, the estimates are imprecise with wide confidence bounds.

#### Rash

There were no consistent associations between skin rash and *Enterococcus* qPCR CE or *Enterococcus* CFU exposures across the age groups, exposures and sites (see S18 and S19 Figs).

### Swimming exposure

**Fig 9:**
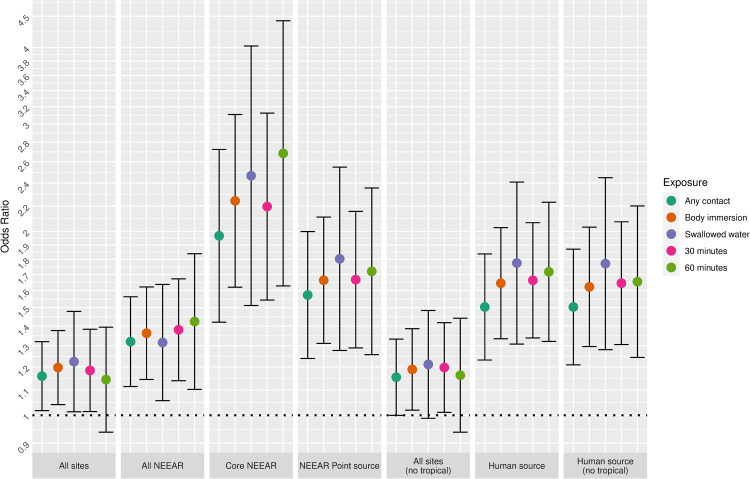
Odds ratios for diarrhea among swimmers associated with a 1-log_10_ increase in Enterococcus qPCR CE among children 10 and under.

Fig 9 highlights the associations between swimming exposures and water quality, focusing on the diarrhea and *Enterococcus* qPCR CE among children 10 and under as an example. For many of the sites, more intense exposures (e.g., swallowing water and spending at least 60 minutes in the water) had the highest odds ratios, whereas for the less intense exposure categories, any contact and body immersion, the associations were attenuated.

#### Site

**Fig 10:**
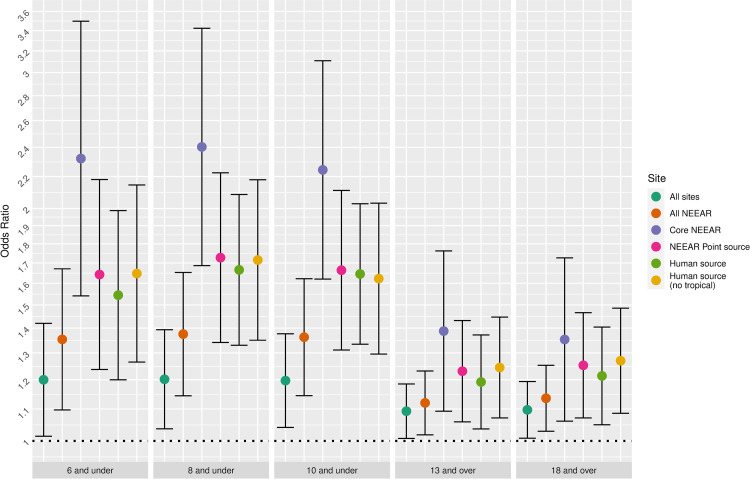
Odds ratios for diarrhea associated with and 1 log10 increase in Enterococcus qPCR CE among body immersion swimmers.

Fig 10 highlights the impact of the type of site on the association between *Enterococcus* qPCR CE and diarrhea for several different age categories. Sites impacted by human sources and the core NEEAR sites tended to have higher risks than sites that were not impacted by human sources. Associations at sites that lacked known human sources of fecal contamination had weaker associations between *Enterococcus* CFU and qPCR CE for the majority of the exposure categories and age groups (Fig 10), even among younger children and for more intense exposures.

## Discussion

In this analysis, we used the largest available dataset on health effects among beachgoers, representing over 80,000 observations from 13 beach sites across the United States and Puerto Rico, to compare associations between acute illnesses and water quality measured by the fecal indicator bacteria, *Enterococcus*, across a range of different age groups, health endpoints, exposures and sites.

These results confirm the previously reported associations between *Enterococcus* exposures and gastrointestinal illness, but also provide evidence that this association is affected by site characteristics, exposure intensities and age. The results highlight four insights regarding the risks associated with swimming in fecally-contaminated recreational waters. First, gastrointestinal symptoms were most consistently associated with fecal contamination across all age groups, exposures and sites, but respiratory symptoms were also associated with fecal contamination among young children. Second, under certain exposure scenarios, children were at higher risk of swimming-associated illness compared to older adolescents and adults 18 and over. Third, the associations between levels of fecal contamination and swimming-associated illness vary depending on the likely sources of fecal contamination and were stronger and more consistent for sites impacted by likely human sources. Fourth, for more intense exposures such as swallowing water and spending over an hour in the water, there was an increased risk of swimming-associated illness compared to less intense exposure categories.

**Fig 11:**
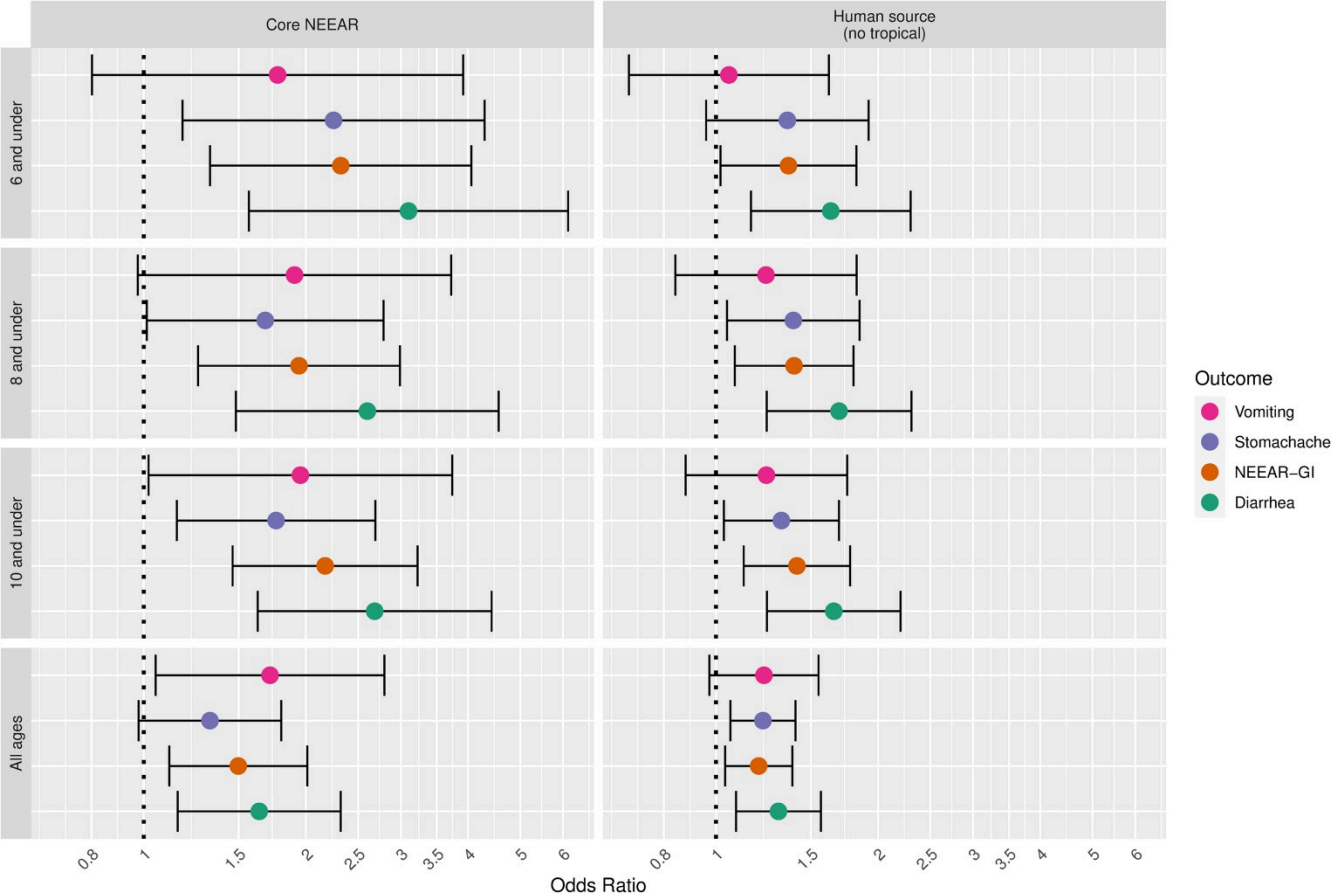
Odds ratios for gastrointestinal outcomes associated with a 1-log10 increase in Enterococcus qPCR CE among those spending 60 minutes or more in the water.

**Fig 12:**
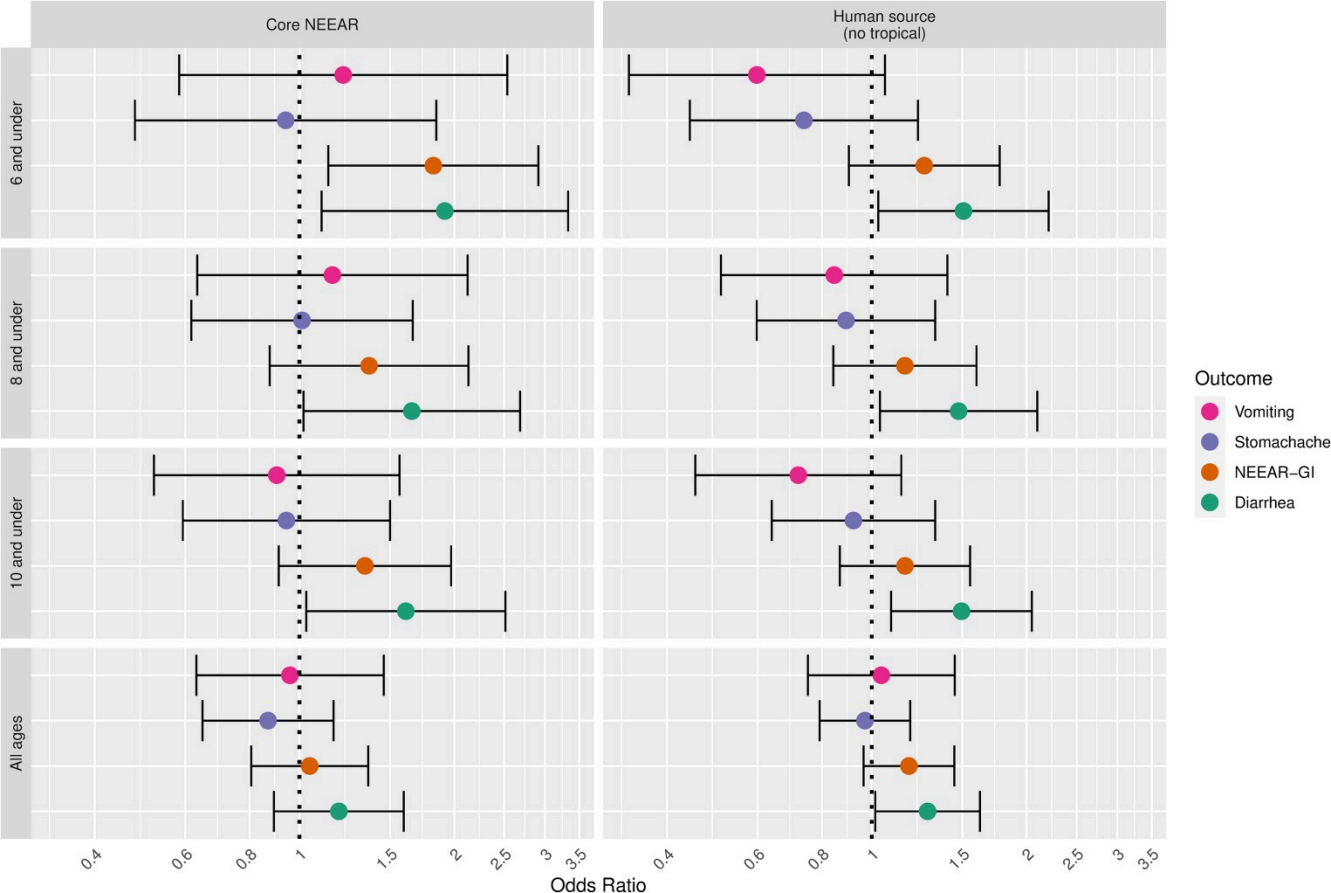
Odds ratios for gastrointestinal outcomes associated with a 1-log10 increase in *Enterococcus* CFU among those spending 60 minutes or more in the water.

Gastrointestinal infections and their associated symptoms have typically been considered the most common health effects resulting from exposure to fecal contamination in recreational waters, particularly when human sewage is the source. A wide range of potentially pathogenic microorganisms found in human sewage can cause gastrointestinal symptoms including: Norovirus, rotavirus, adenovirus, *Cryptosporidium* spp., *Giardia lamblia*, *Campylobacter jejuni*, *Salmonella enterica*, and *Escherichia coli* O157:H7. These infections cause a range of gastrointestinal symptoms, but in this study, diarrhea, showed somewhat stronger associations with fecal contamination compared to the composite NEEAR-GI Illness definition and other gastrointestinal symptoms (see Figs 11 and 12). For example, at the core NEEAR sites, among children 6 and under who spent 60 minutes in the water, the odds ratio associated for diarrhea associated a log_10_ increase in *Enterococcus* qPCR CE was 3.10 (95% CI 1.57–6.14) and for GI illness for the same exposure scenario was 2.32 (95% CI 1.33–4.06). This trend was also evident for *Enterococcus* CFU (Fig 12). Some individual symptoms such as vomiting, occurred relatively infrequently, resulting in inconsistent and less precise associations.

Associations between illness and *Enterococcus* were stronger among children (defined by various age categories) compared to adults for many exposure scenarios, but it was most apparent for gastrointestinal outcomes at the core NEEAR sites and likely human impacted sites (see Figs 1–4 and 10). Severe gastrointestinal illness was less consistently associated with *Enterococcus* but some very strong associations (odds ratios> 5) were observed among young children with *Enterococcus* qPCR CE (6 and under; 4 and under categories), highlighting the potentially vulnerability of this group to experience more severe illness following infection. The age categories considered in this study were overlapping because there was generally not sufficient data to examine specific narrow age ranges separately. However, for additional comparison and context, we also considered alternate age categories (ages 4–10; 4–12; 6–10; and 6–12, see S1–S3 and S20 Figs). There were no notable differences in risks for the different age categories, but sample sizes were relatively small and effect estimates were imprecise for very young age groups and narrow age ranges.

Respiratory illness and respiratory symptoms were also associated with *Enterococcus* (both qPCR and CFU, see Figs 6–8) at core NEEAR sites and likely human impacted sites among younger children (6 and under and 4 and under) but less so among older children and adults. Studies have observed associations between respiratory illness and fecal contamination of recreational waters [36, 37], but clear differences between children and adults have not been reported. Children are generally more susceptible to respiratory illnesses and may inhale or ingest pathogens that can cause respiratory symptoms such as enteroviruses (coxsackieviruses and echoviruses) and adenoviruses during swimming [8].

Rash was unassociated with levels of *Enterococcus*. Although rash has often been reported to occur at higher rates among swimmers compared to non-swimmers, associations with fecal contamination have been inconsistent [38, 39]. Skin rashes following swimming may often be associated with physical irritation, or caused by microorganisms not directly related to fecal contamination (e.g., cyanobacteria or allergic reactions to avian schistosomes resulting in cercarial dermatitis or “swimmer’s itch”). Infected cuts, which may be associated with fecal contamination were not considered in this study because there were too few cases reported. We also did not consider earaches/ear infections as a previous analysis showed no association with indicators of fecal contamination [40].

Swallowing water and staying in the water at least 60 minutes resulted in the higher risks in many scenarios (Figs 1–5 and 9). These activities imply a prolonged and more intense exposure which may result in the increased exposure to waterborne pathogens and a higher risk of infection.

Sites impacted by human sewage or likely human sources of fecal contamination had the most consistent associations between GI illness and *Enterococcus* exposures, confirming that the source of fecal contamination is an important factor to consider in risk characterization and management. Risks of illness associated with *Enterococcus* were highest at the core NEEAR sites. One possible explanation for this observation is that the core NEEAR sites were specifically selected to be impacted by wastewater discharges from source populations of at least 15,000 individuals. As first discussed by Cabelli et al.[41], small discharging source populations can increase variability in the ratios between indicators and pathogens, and as a result, may impact associations between indicator levels and health effects.

### Considerations and limitations

This study focused on associations between *Enterococcus* exposure and illness risk among swimmers (swimming exposure defined in several different ways), expressed as odds ratios. We did not calculate risk differences, or swimming-associated illness rates relative to non-swimmers (or baseline risk). The risk difference measure is commonly used to assess acceptable or tolerable risk ranges and set guideline values (e.g., 32 excess cases of GI illness per 1000 swimmers). However, this estimate can be affected by the selection of the reference group and the associated baseline risk. The reference group and thus the baseline risk will vary by age and site categories, making associations difficult to compare. This analysis focused on odds ratios among swimmers to provide an approximately common estimate and scale to compare the relative magnitude of the associations across the varying categories of health endpoint, age, exposure, and site, independent of the baseline risk. In addition, because non-swimmers likely represent a distinctly different population (for example individuals may choose not to swim because they were recently ill, or are in poorer general health), by restricting analysis to swimmers the potential for uncontrolled confounding or bias was reduced. Finally, restricting the analysis to swimmers only likely reduced potential differential recall of illness resulting from participants awareness of their swimming exposure (i.e., lack of blinding to swimming status).

The predominant type of fecal contamination impacting beach sites can vary based on local conditions. For example, at human impacted sites, dogs, birds and runoff can contribute non-human fecal contamination. At sites predominantly impacted by non-human sources, stormwater, swimmers and beach goers themselves can be a source of human fecal contamination [42]. We classified sites as human impacted and not human impacted based on the best available information and previous studies available for each site. We recognize that this binary classification is an oversimplification of a complex system, which may result in misclassification errors for particular days, but nonetheless believe it is a useful stratification as these sources have been hypothesized to present distinctly different health risks [28, 43].

Even at sites impacted by human sources, the source of contamination can impact health risk. For example, raw sewage from leaking infrastructure (sewage or septic) or runoff and effluent discharge from wastewater treatment plants will likely differ in terms of their densities of indicator bacteria and the types of pathogens present. Wastewater treatment is usually effective at removing bacteria and bacterial indicators, but may allow the survival of pathogens such as enteric viruses (e.g., noroviruses) or other pathogens that may have some resistance to disinfection[44]. We have previously hypothesized that this may be why we observed a stronger association between *Enterococcus* measured by qPCR (which may still detect DNA following cell inactivation or death [45]) and gastrointestinal symptoms than *Enterococcus* measured by culture[16]. Ultimately, however, we lack the data to fully address these complexities in this analysis.

We conducted numerous different analyses and made many comparisons, so some statistically significant associations will occur by chance alone. As a result, p-values and significance levels (i.e., confidence bounds that cross or exclude the no-effect value of 1 for odds ratios) should not be interpreted as strict acceptance or rejection of any null hypotheses. Rather, we have interpreted the results according to the magnitude, consistency and the precision of the effect estimates. We also conducted statistical tests for interaction by age, and highlighted those that were statistically significant (p<0.05). However, tests for interaction can have low statistical power [46], and this was particularly likely for some of the smaller subsets (e.g., rarer outcome such as severe gastrointestinal illness and less frequent exposures such as swallowing water).

We used the daily averaged *Enterococcus* densities to assign exposure to swimmers, combining estimates across time and location on any given day. Because levels of *Enterococcus* and other fecal indicator bacteria vary over time and space [47], this may have resulted in misclassification of exposure to *Enterococcus*. However, we expect this misclassification would be unbiased with regard to the health outcomes. We have also previously demonstrated that daily average densities are as good or better at predicting health effects than time or location specific estimates [16, 18].

## Conclusion

This study found that under many exposure scenarios, children, defined using several different age groupings, were at higher risk of swimming-associated illness associated with the fecal indicator bacteria *Enterococcus* in recreational waters. Gastrointestinal symptoms were the most sensitive endpoint and respiratory symptoms were also associated with *Enterococcus* levels among children. The source of fecal contamination and the intensity of swimming exposure were also important factors affecting the association between *Enterococcus* and swimming-associated illness.

## Supporting information

S1 Table Site descriptions. (DOCX)

S2 Table Site classifications. (DOCX)

S3 Table Study population. (DOCX)

S4 Table Water quality- daily geometric means (per 100 ml). (DOCX)

S1 Fig Odds ratios for NEEAR-GI associated with a 1 log_10_ increase in *Enterococcus* qPCR CE, alternate age categories. (PDF)

S2 Fig Odds ratios for NEEAR-GI associated with a 1 log_10_ increase in *Enterococcus* CFU, alternate age categories. (PDF)

S3 Fig Odds ratios for diarrhea associated with a 1 log_10_ increase in *Enterococcus* qPCR CE, alternate age categories. (PDF)

S4 Fig Odds ratios for severe gastrointestinal illness associated with a 1 log_10_ increase in *Enterococcus* CFU. (PDF)

S5 Fig Odds ratios for vomiting associated with a 1 log_10_ increase in *Enterococcus* qPCR CE. (PDF)

S6 Fig Odds ratios for nausea associated with a 1 log_10_ increase in *Enterococcus* qPCR CE. (PDF)

S7 Fig Odds ratios for stomachache associated with a 1 log_10_ increase in *Enterococcus* qPCR CE. (PDF)

S8 Fig Odds ratios for vomiting associated with a 1 log_10_ increase in *Enterococcus* CFU. (PDF)

S9 Fig Odds ratios for nausea associated with a 1 log_10_ increase in *Enterococcus* CFU. (PDF)

S10 Fig Odds ratios for stomachache associated with a 1 log_10_ increase in *Enterococcus* CFU. (PDF)

S11 Fig Odds ratios for sore throat associated with a 1 log_10_ increase in *Enterococcus* qPCR CE. (PDF)

S12 Fig Odds ratios for cough associated with a 1 log_10_ increase in *Enterococcus* qPCR CE. (PDF)

S13 Fig Odds ratios for cold associated with a 1 log_10_ increase in *Enterococcus* qPCR CE. (PDF)

S14 Fig Odds ratios for respiratory illness associated with a 1 log_10_ increase in *Enterococcus* CFU. (PDF)

S15 Fig Odds ratios for sore throat associated with a 1 log_10_ increase in *Enterococcus* CFU. (PDF)

S16 Fig Odds ratios for cough associated with a 1 log_10_ increase in *Enterococcus* CFU. (PDF)

S17 Fig Odds ratios for cold associated with a 1 log_10_ increase in *Enterococcus* CFU. (PDF)

S18 Fig Odds ratios for rash associated with a 1 log_10_ increase in *Enterococcus* qPCR CE. (PDF)

S19 Fig Odds ratios for rash associated with a 1 log_10_ increase in *Enterococcus* CFU. (PDF)

S20 Fig Odds ratios for diarrhea associated with a 1 log_10_ increase in *Enterococcus* CFU, alternate age categories. (PDF)
