# Correction: Health risks to children from exposure to fecally-contaminated recreational water

**DOI:** 10.1371/journal.pone.0315836

**Published:** 2024-12-12

**Authors:** Timothy J. Wade, Benjamin F. Arnold, Ken Schiff, John M. Colford, Stephen B. Weisberg, John F. Griffith, Alfred P. Dufour

There is an error in S2 Table. The site Boquerón Beach should not be included in the “NEEAR-core sites” category. Please see the correct S2 Table here.

**S2 Table:** Site classifications.

Category	Sites	
All sites	Avalon Beach Boquerón Beach Doheny Beach Edgewater Beach Fairhope Beach Goddard State Park Beach Huntington Beach	Malibu Beach Mission Bay Silver Beach Surfside Beach Washington Park Beach West Beach
Human impacted	Avalon Beach (ground water discharge above median)^b^ Boquerón Beach Doheny Beach (berm open)^a^ Edgewater Beach Fairhope Beach	Goddard State Park Beach Huntington Beach Washington Park Beach West Beach Silver Beach
Human impacted (excluding tropical)	Avalon Beach (ground water discharge above median)^b^ Doheny Beach (berm open)^a^ Edgewater Beach Fairhope Beach	Goddard State Park Beach Huntington Beach Washington Park Beach West Beach Silver Beach
All NEEAR	Boquerón Beach Edgewater Beach Fairhope Beach Goddard State Park Beach Huntington Beach	Silver Beach Surfside Beach Washington Park Beach West Beach
All NEEAR- point source	Boquerón Beach Edgewater Beach Fairhope Beach Goddard State Park Beach	Huntington Beach Silver Beach Washington Park Beach West Beach
NEEAR- core sites	Edgewater Beach Fairhope Beach Goddard State Park Beach Huntington Beach	Silver Beach Washington Park Beach West Beach
