# Supplementary material for: Health risks to children from exposure to fecally-contaminated recreational water
Source: PLoS One. 2022 Apr 12;17(4):e0266749. doi: 10.1371/journal.pone.0266749 (PMC9004770; doi:10.1371/journal.pone.0266749)
Supplement: S1 Table — (DOCX) [file pone.0266749.s001.docx]

S1 Table. Site descriptions

| **Beach** | **Location** | **Year** | **Notes** | **Number enrolled** |
| --- | --- | --- | --- | --- |
| West Beach | Indiana Dunes State Park, Lake Michigan | 2003 | Freshwater, Great Lakes | 2,877 |
| Huntington Beach | Bay Village Ohio, Lake Erie | 2003 | Freshwater, Great Lakes | 2,840 |
| Silver Beach | St. Joseph Michigan, Lake Michigan | 2004 | Freshwater, Great Lakes | 10,921 |
| Washington Park Beach | Michigan City Indiana, Lake Michigan | 2004 | Freshwater, Great Lakes | 4,377 |
| Edgewater Beach | Edgewater, Mississippi | 2005 | Marine, Gulf Coast | 1,351 |
| Fairhope Beach | Fairhope, Alabama | 2007 | Marine, Gulf Coast | 2,022 |
| Goddard State Park Beach | Goddard State Park, Rhode Island | 2007 | Marine, Atlantic | 2,977 |
| Surfside Beach | Surfside, South Carolina | 2009 | Marine, Atlantic | 11,159 |
| Boquerón Beach | Boquerón, Puerto Rico | 2009 | Marine, Atlantic, Tropical | 15,726 |
| Malibu Beach | Malibu, California | 2009 | Marine, Atlantic | 5,674 |
| Doheny Beach | Dana Point, California | 2007-2008 | Marine, Pacific | 9,303 |
| Mission Bay | San Diego, California | 2003 | Marine, Pacific | 8,076 |
| Avalon Beach | Catalina Island, California | 2007-2008 | Marine, Pacific | 6,149 |
