# Supplementary material for: Health risks to children from exposure to fecally-contaminated recreational water
Source: PLoS One. 2022 Apr 12;17(4):e0266749. doi: 10.1371/journal.pone.0266749 (PMC9004770; doi:10.1371/journal.pone.0266749)
Supplement: S2 Table — (DOCX) [file pone.0266749.s002.docx]

S2 Table. Site classifications

| Category | Sites | |
| --- | --- | --- |
| All sites | Avalon Beach  Boquerón Beach  Doheny Beach  Edgewater Beach  Fairhope Beach  Goddard State Park Beach  Huntington Beach | Malibu Beach  Mission Bay  Silver Beach  Surfside Beach  Washington Park Beach  West Beach |
| Human impacted | Avalon Beach (ground water discharge above median)^b^  Boquerón Beach  Doheny Beach (berm open)^a^  Edgewater Beach  Fairhope Beach | Goddard State Park Beach  Huntington Beach  Washington Park Beach  West Beach  Silver Beach |
| Human impacted (excluding tropical) | Avalon Beach (ground water discharge above median)^b^  Doheny Beach (berm open)^a^  Edgewater Beach  Fairhope Beach | Goddard State Park Beach  Huntington Beach  Washington Park Beach  West Beach  Silver Beach |
| All NEEAR | Boquerón Beach  Edgewater Beach  Fairhope Beach  Goddard State Park Beach  Huntington Beach | Silver Beach  Surfside Beach  Washington Park Beach  West Beach |
| All NEEAR- point source | Boquerón Beach  Edgewater Beach  Fairhope Beach  Goddard State Park Beach | Huntington Beach  Silver Beach  Washington Park Beach  West Beach |
| NEEAR- core sites | Boquerón Beach  Edgewater Beach  Fairhope Beach  Goddard State Park Beach | Huntington Beach  Silver Beach  Washington Park Beach  West Beach |

^a: For discussion see: Colford JM, Jr., Schiff KC, Griffith JF, Yau V, Arnold BF, Wright CC, et al. Using rapid indicators for Enterococcus to assess the risk of illness after exposure to urban runoff contaminated marine water. Water Res. 2012;46(7):2176-86.^

^b: For discussion see: Yau VM, Schiff KC, Arnold BF, Griffith JF, Gruber JS, Wright CC, et al. Effect of submarine groundwater discharge on bacterial indicators and swimmer health at Avalon Beach, CA, USA. Water Res. 2014;59:23-36.^
