# Supplementary material for: Health risks to children from exposure to fecally-contaminated recreational water
Source: PLoS One. 2022 Apr 12;17(4):e0266749. doi: 10.1371/journal.pone.0266749 (PMC9004770; doi:10.1371/journal.pone.0266749)
Supplement: S3 Table — (DOCX) [file pone.0266749.s003.docx]

S3 Table. Study population

|  | **All sites** | **Human sources** | **Human sources (excluding tropical)** | **All NEEAR** | **All NEEAR- point source** | **NEEAR- core sites** |
| --- | --- | --- | --- | --- | --- | --- |
| Total | 83,452 | 48,550 | 45,539 | 54,250 | 43,091 | 27,365 |
| *Age groups* |  |  |  |  |  |  |
| 12 and under | 20,458 | 11,229 | 10,447 | 11,728 | 9,619 | 6,632 |
| 10 and under | 17,134 | 9,371 | 8,730 | 9,769 | 8,017 | 5,636 |
| 6 and under | 9,907 | 5,450 | 6,918 | 5,681 | 4,699 | 3,364 |
| 4 and under | 6,494 | 3,585 | 3,383 | 3,761 | 3,114 | 2,206 |
| *Exposure categories* |  |  |  |  |  |  |
| Any water contact | 58,195 | 33,655 | 31,483 | 39,544 | 30,186 | 17,571 |
| Body immersion swimmers | 47,036 | 27,912 | 26,031 | 33,264 | 25,191 | 13,080 |
| Swallowed Water | 10,787 | 5,664 | 5,238 | 7,113 | 5,010 | 2,378 |
| Water- 30 minutes | 36,777 | 13,356 | 19,969 | 24,748 | 19,248 | 8,475 |
| Water- 60 minutes | 22,898 | 21,361 | 12,704 | 15,190 | 12,307 | 4,303 |
| *Illness* |  |  |  |  |  |  |
| GI illness | 5,629 | 3,315 | 3,058 | 3,589 | 2,884 | 2,101 |
| Diarrhea | 3,872 | 2,221 | 2,026 | 2,400 | 1,920 | 1,410 |
| Severe GI illness | 1,211 | 779 | 703 | 798 | 662 | 442 |
| Respiratory illness | 4,880 | 3,158 | 3,012 | 3,456 | 2,887 | 1,681 |
| Sore throat | 4,586 | 2,963 | 2,809 | 3,165 | 2,685 | 1,520 |
| Cough | 2,597 | 1,550 | 1,474 | 1,681 | 1,399 | 740 |
| Cold | 3,422 | 2,368 | 2,254 | 2,585 | 2,163 | 1,241 |
| Rash | 3,060 | 1,784 | 1,687 | 2,089 | 1,614 | 861 |
