# Supplementary material for: Health risks to children from exposure to fecally-contaminated recreational water
Source: PLoS One. 2022 Apr 12;17(4):e0266749. doi: 10.1371/journal.pone.0266749 (PMC9004770; doi:10.1371/journal.pone.0266749)
Supplement: S4 Table — (DOCX) [file pone.0266749.s004.docx]

S4 Table 4. Water quality- daily geometric means (per 100 ml)

|  | **All sites** | **Human sources** | **Human sources (excluding tropical)** | **All NEEAR** | **All NEEAR- point source** | **NEEAR- core sites** |
| --- | --- | --- | --- | --- | --- | --- |
| *Enterococcus CFU* |  |  |  |  |  |  |
| N | 360 | 212 | 183 | 203 | 174 | 148 |
| Min | 0.1 | 0.1 | 0.3 | 0.3 | 0.29 | 0.29 |
| 25^th^ % | 3 | 4 | 4 | 3 | 4 | 5 |
| Median | 10 | 14 | 12 | 10 | 12 | 14 |
| Mean | 41 | 40 | 37 | 27 | 30 | 34 |
| 75^th^ % | 29 | 30 | 28 | 27 | 28 | 31 |
| Max | 3100 | 1357 | 1357 | 1042 | 1042 | 1042 |
| *Enterococcus qPCR CE* |  |  |  |  |  |  |
| N | 347 | 212 | 183 | 203 | 174 | 148 |
| Min | 0.1 | 0.23 | 0.3 | 0.15 | 0.23 | 2 |
| 25^th^% | 10 | 18 | 16 | 15 | 17 | 28 |
| Median | 38 | 66 | 59 | 59 | 66 | 83 |
| Mean | 137 | 152 | 146 | 175 | 150 | 174 |
| 75^th^% | 141 | 187 | 180 | 171 | 188 | 222 |
| Max | 7317 | 1421 | 1421 | 7317 | 1420 | 1421 |
