# Supplementary figures and images for: Health risks to children from exposure to fecally-contaminated recreational water
Source: PLoS One. 2022 Apr 12;17(4):e0266749. doi: 10.1371/journal.pone.0266749 (PMC9004770; doi:10.1371/journal.pone.0266749)

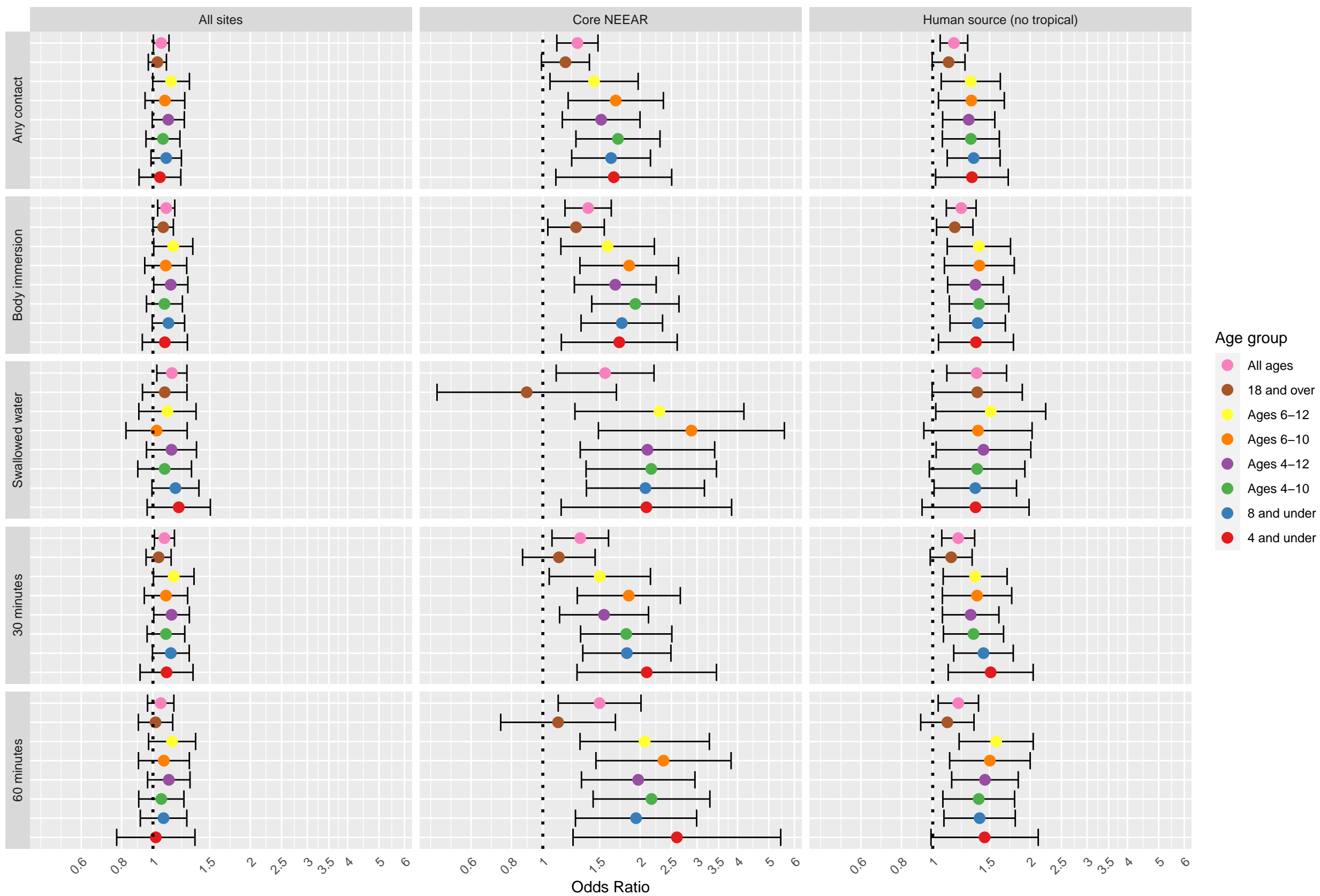

Supplement: S1 Fig — (PDF) [file pone.0266749.s005.pdf]

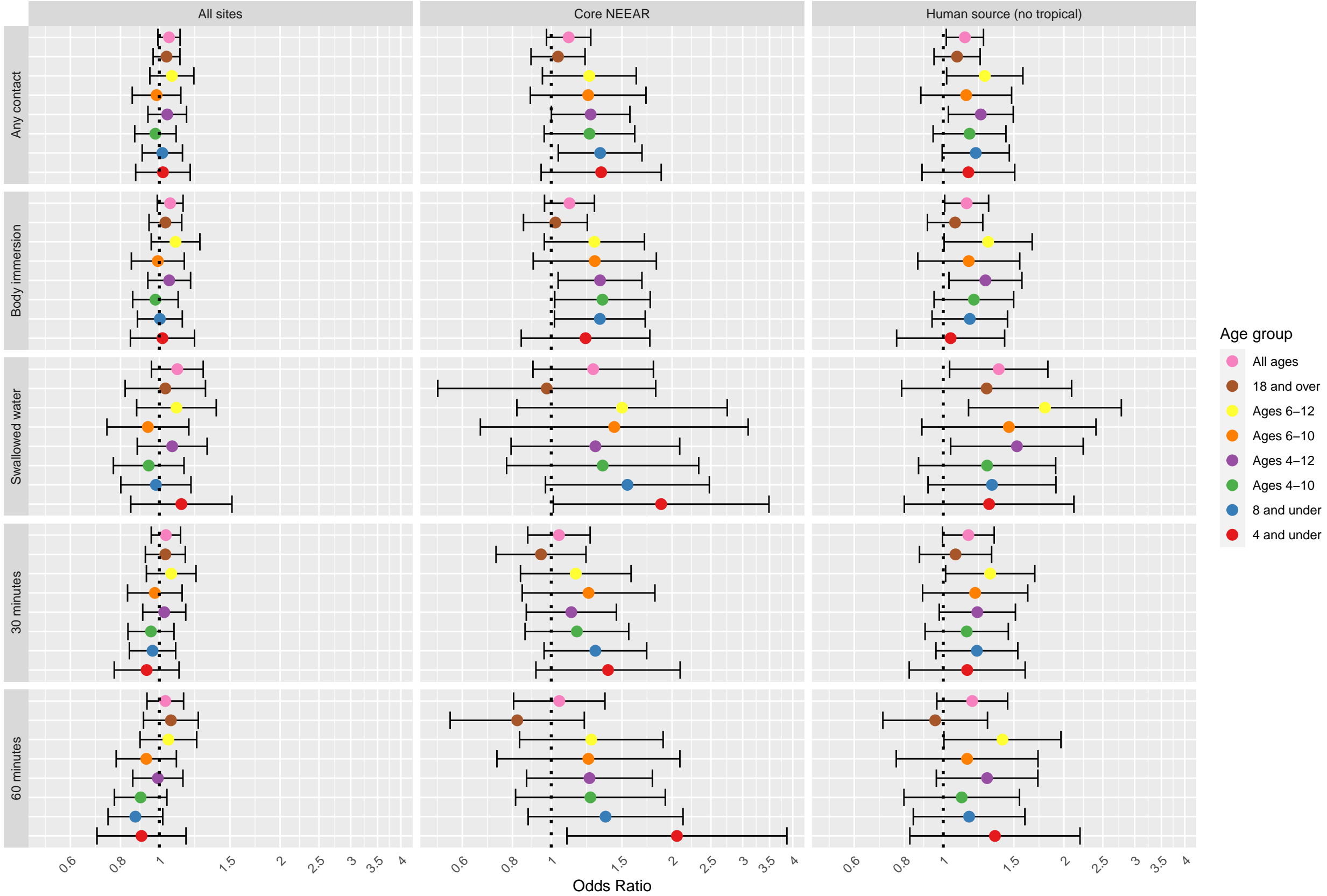

Supplement: S2 Fig — (PDF) [file pone.0266749.s006.pdf]

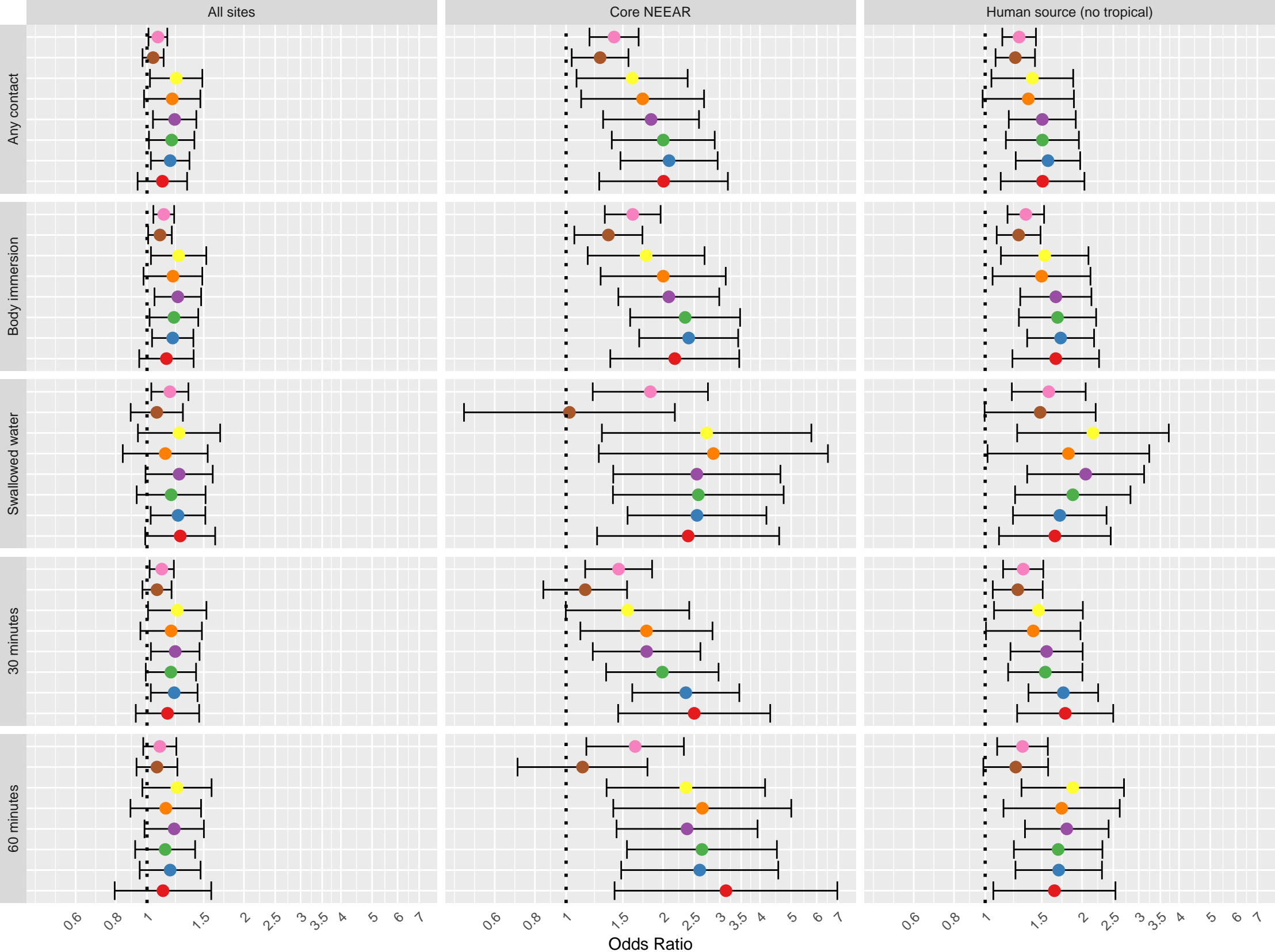

Supplement: S3 Fig — (PDF) [file pone.0266749.s007.pdf]

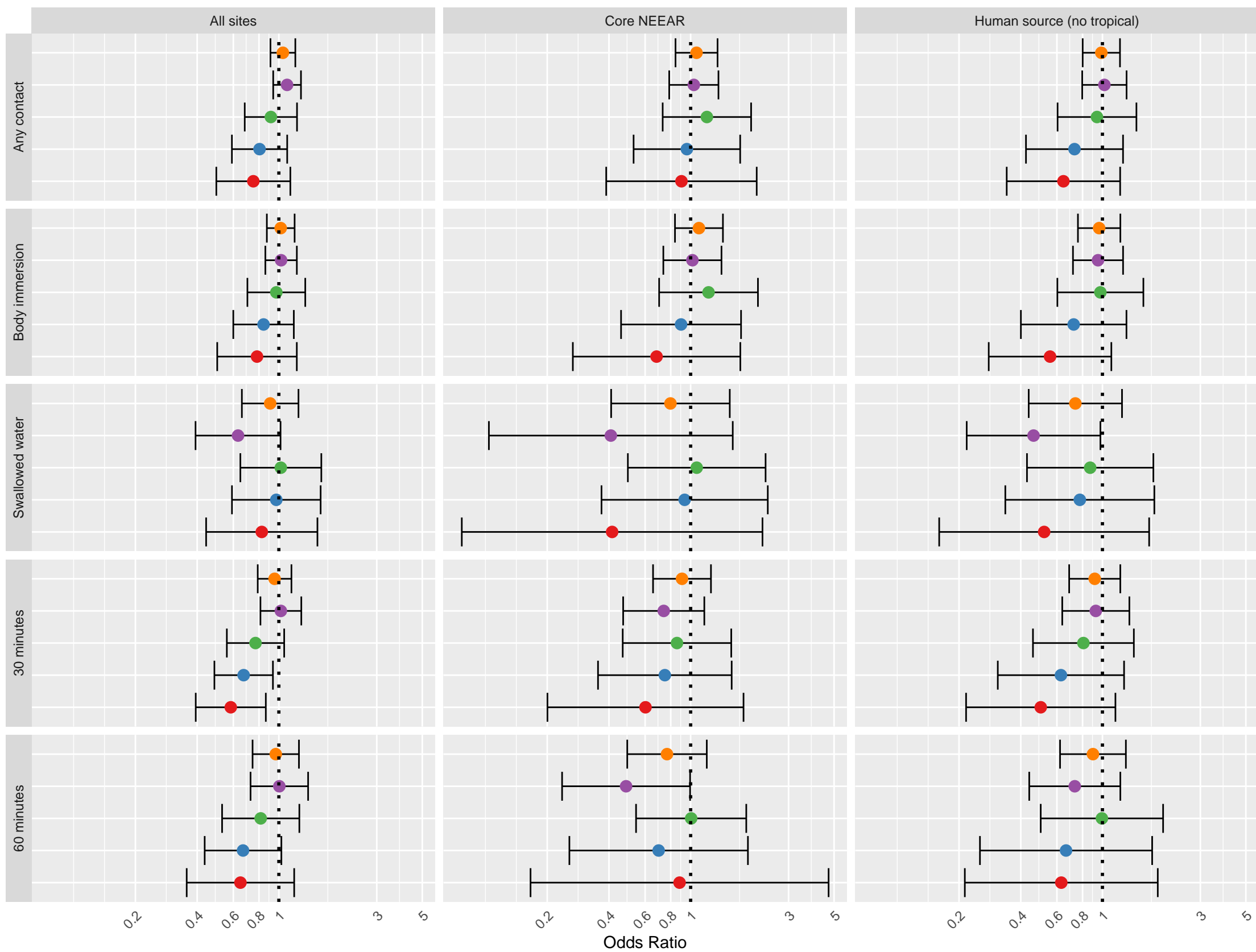

Supplement: S4 Fig — (PDF) [file pone.0266749.s008.pdf]

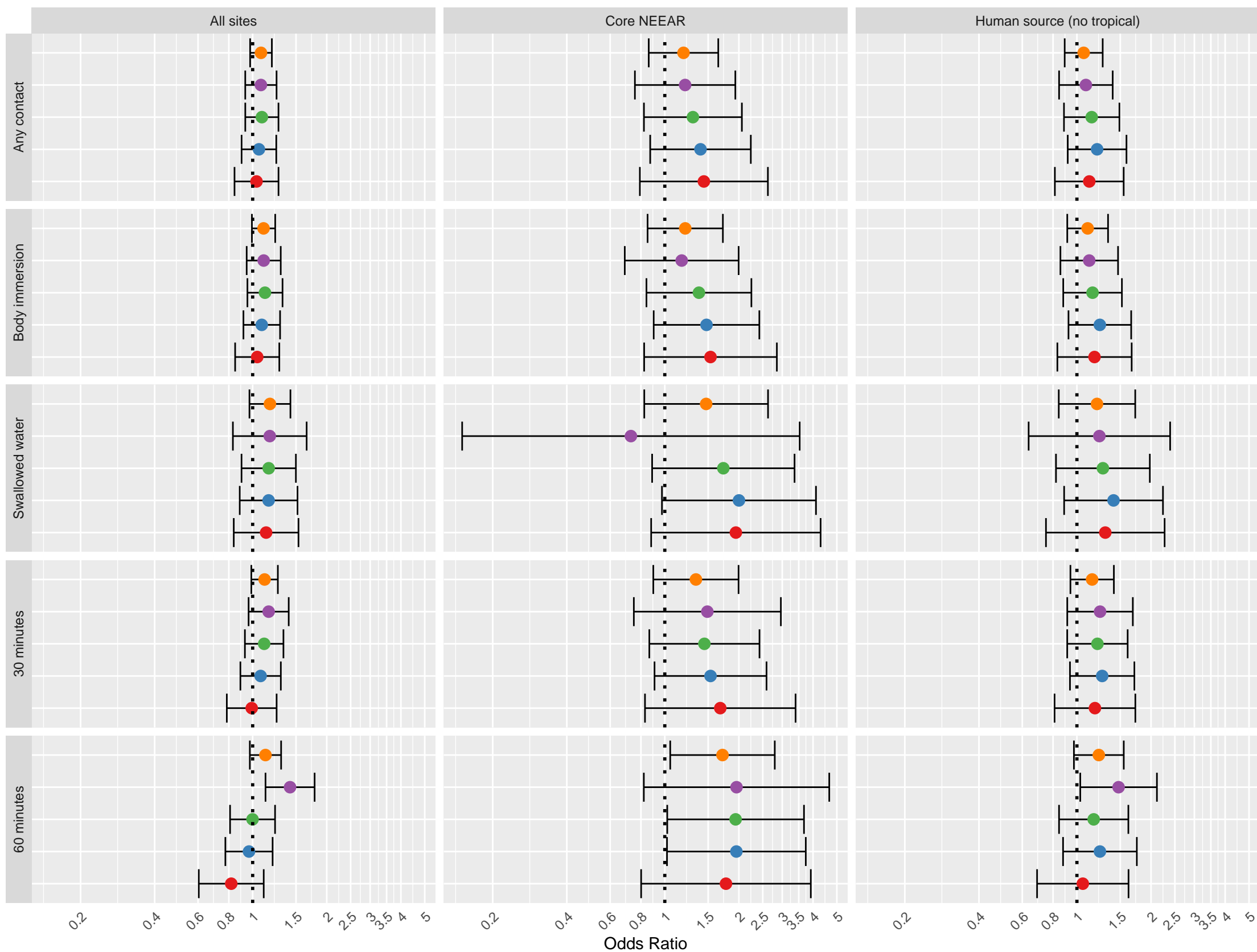

Supplement: S5 Fig — (PDF) [file pone.0266749.s009.pdf]

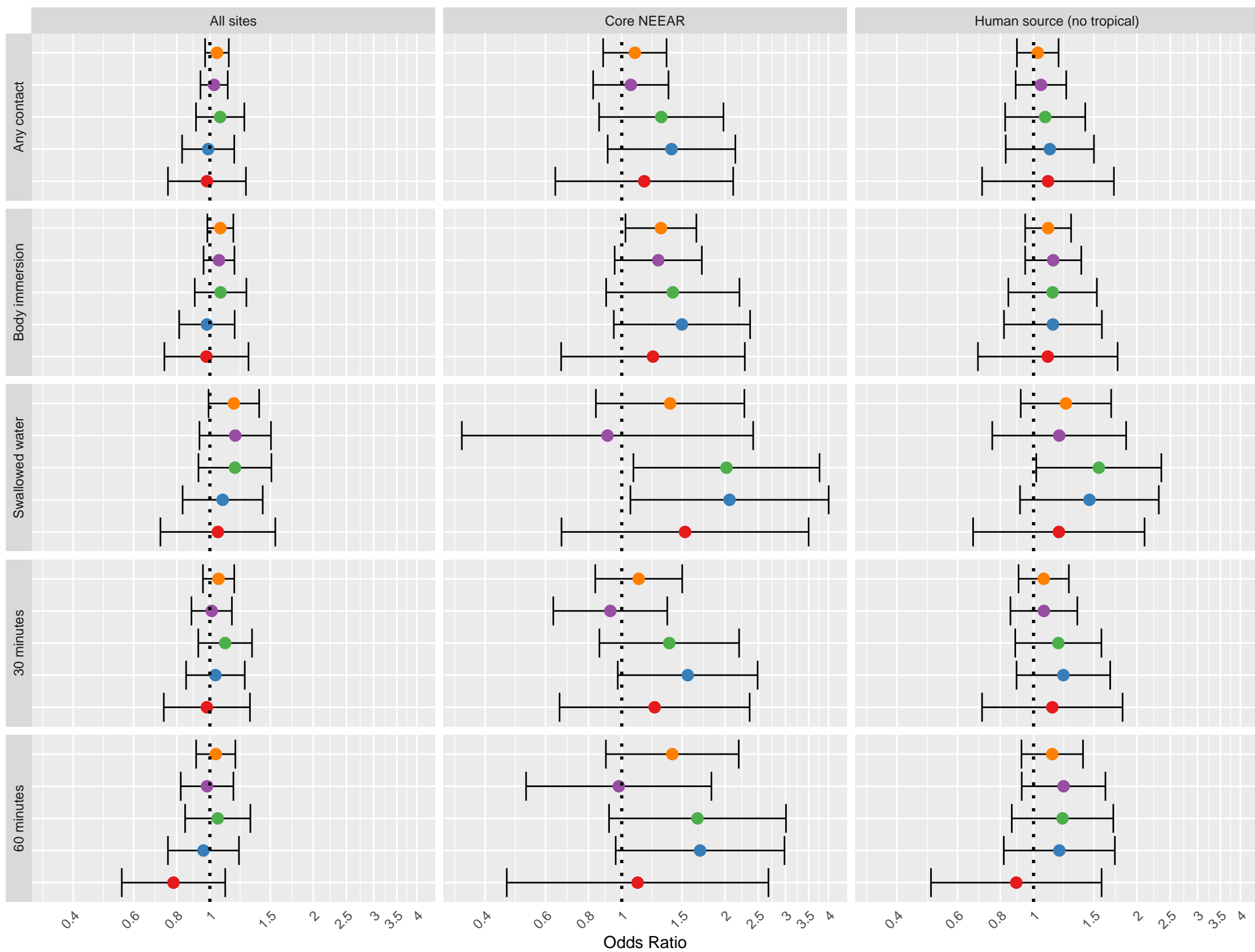

Supplement: S6 Fig — (PDF) [file pone.0266749.s010.pdf]

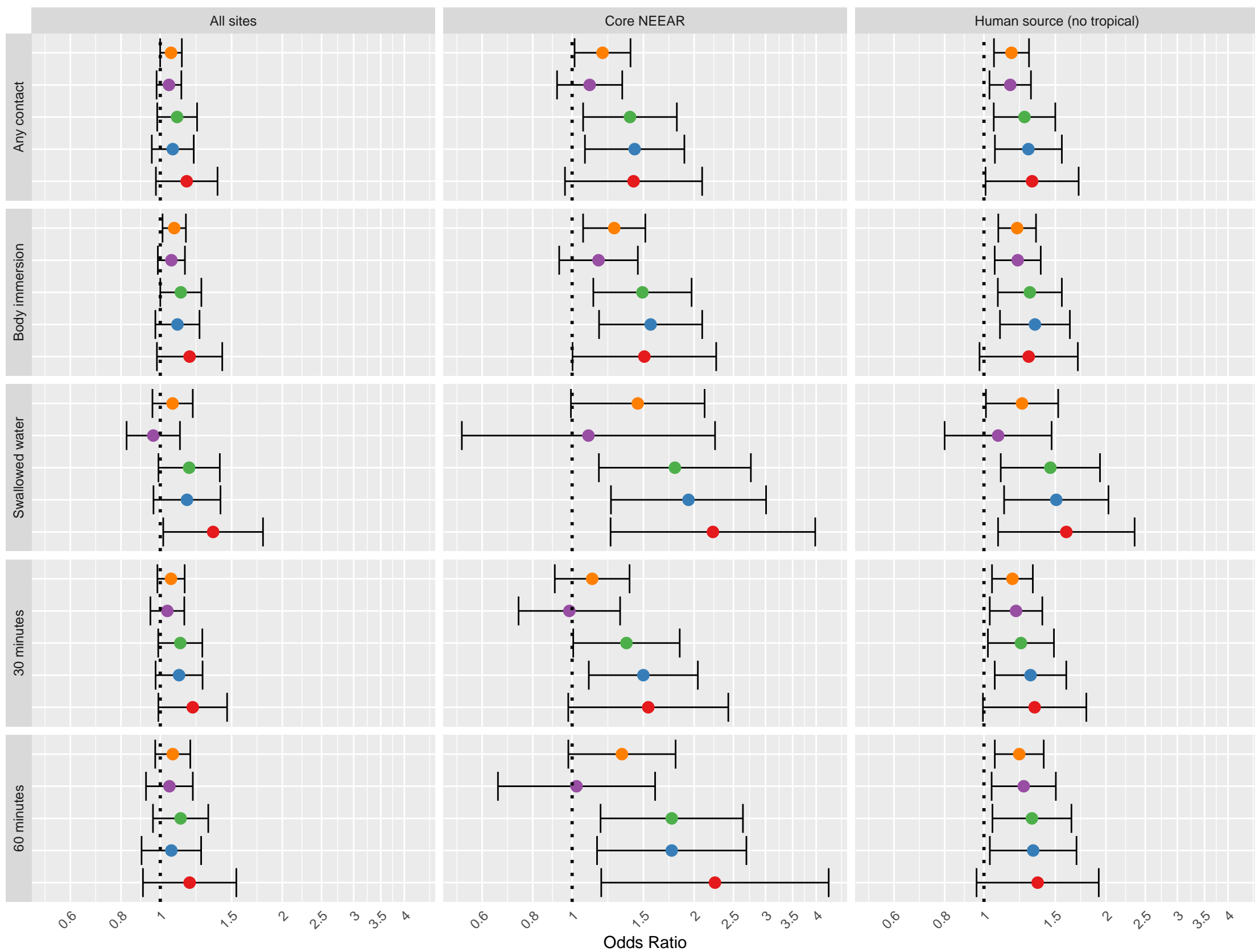

Supplement: S7 Fig — (PDF) [file pone.0266749.s011.pdf]

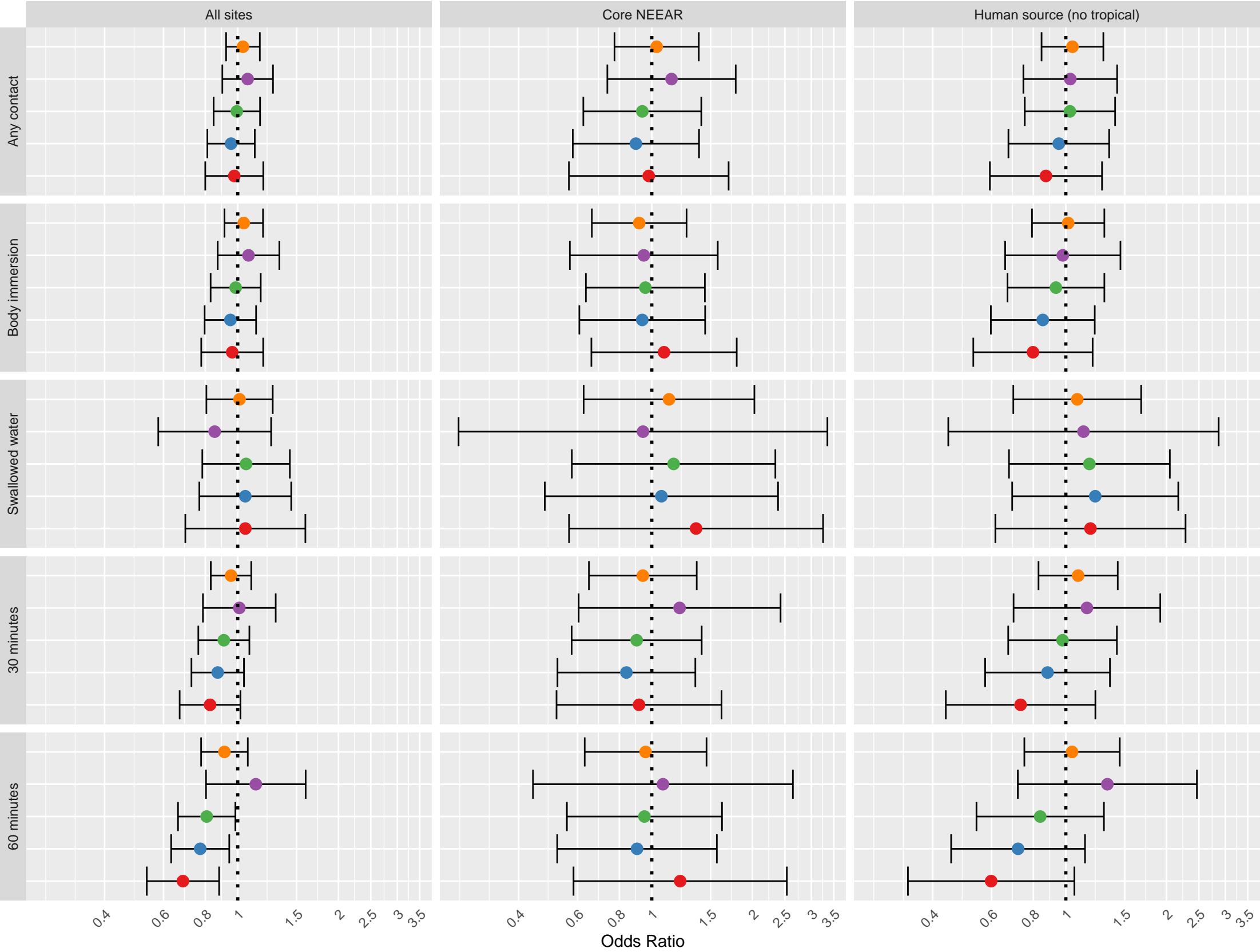

Supplement: S8 Fig — (PDF) [file pone.0266749.s012.pdf]

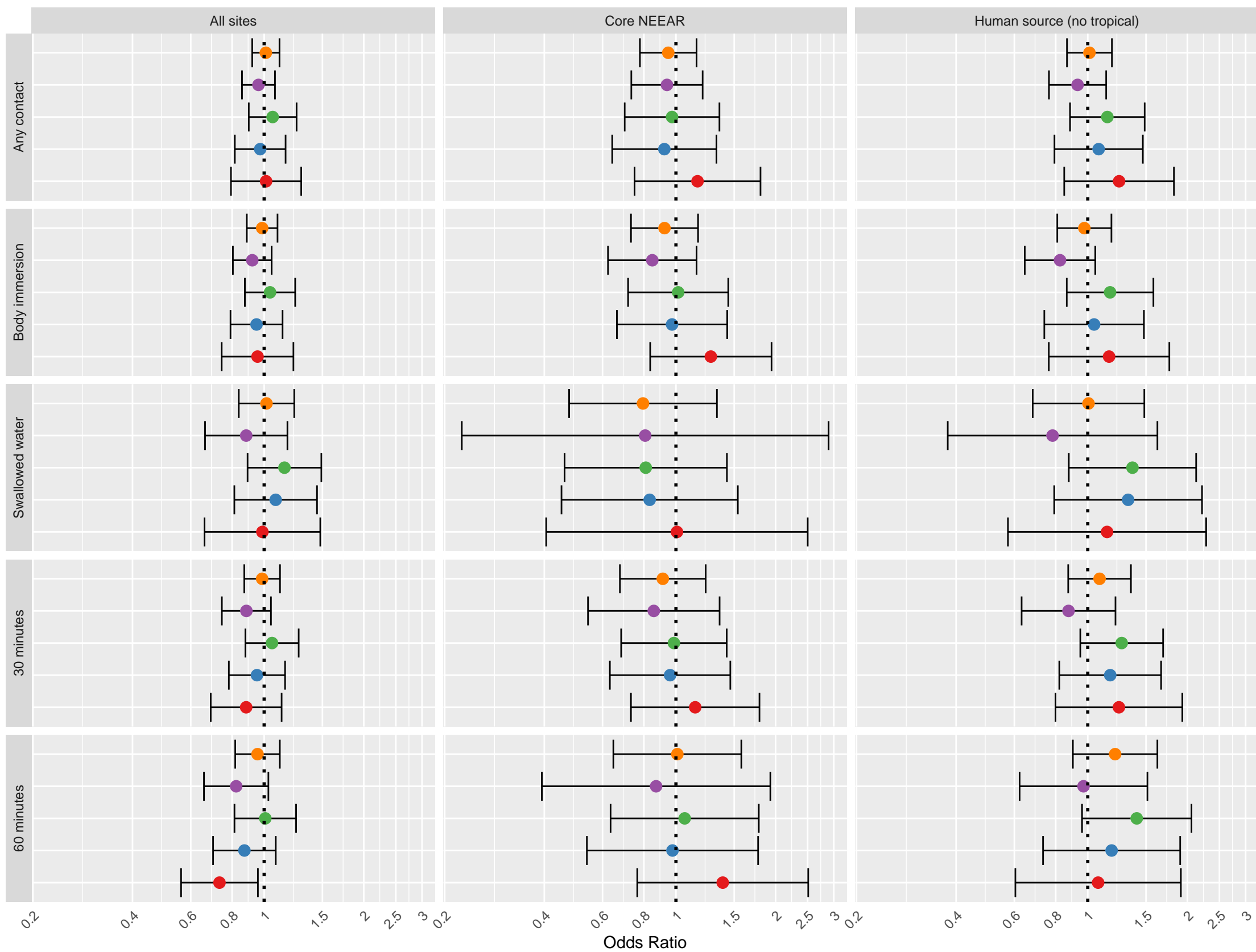

Supplement: S9 Fig — (PDF) [file pone.0266749.s013.pdf]

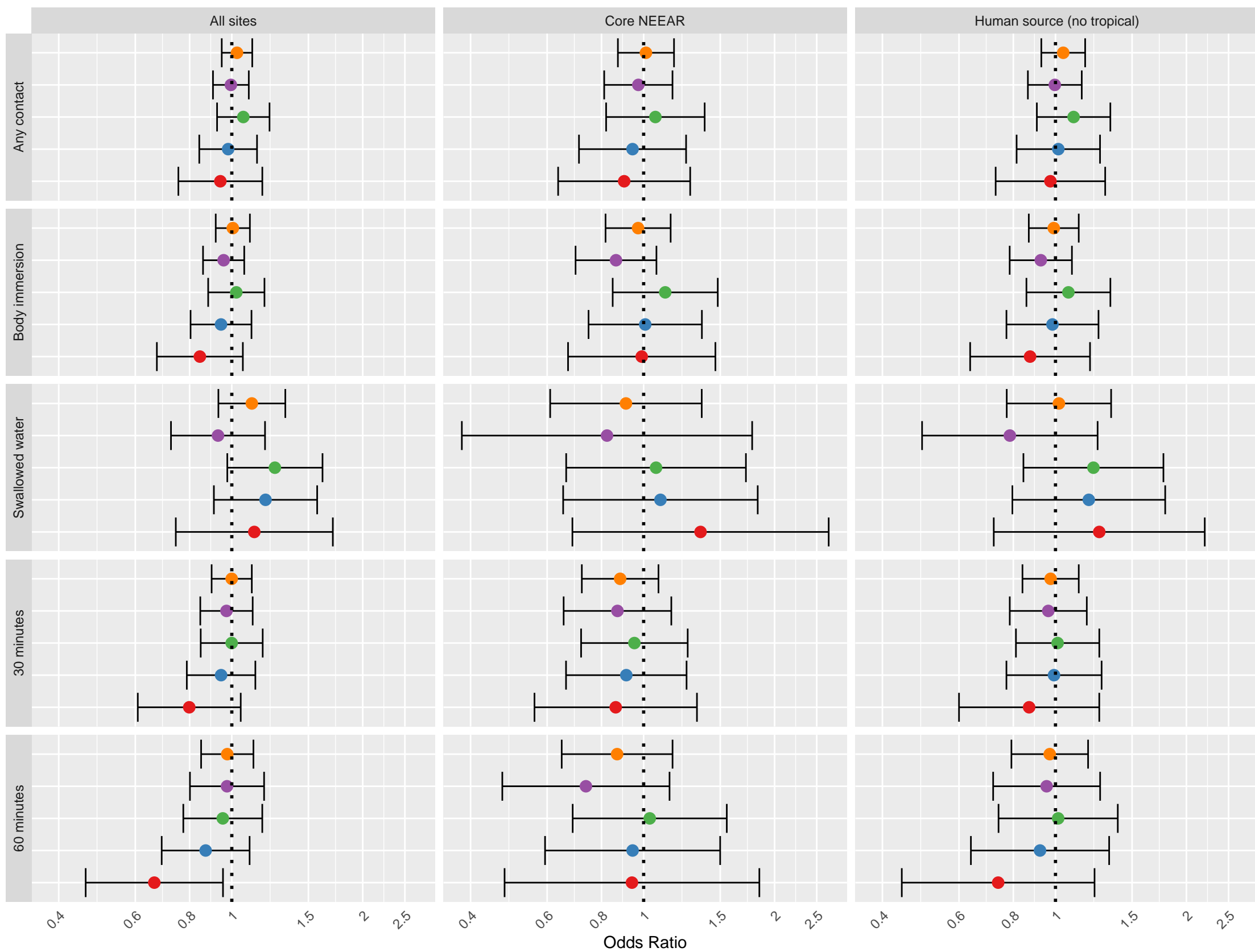

Supplement: S10 Fig — (PDF) [file pone.0266749.s014.pdf]

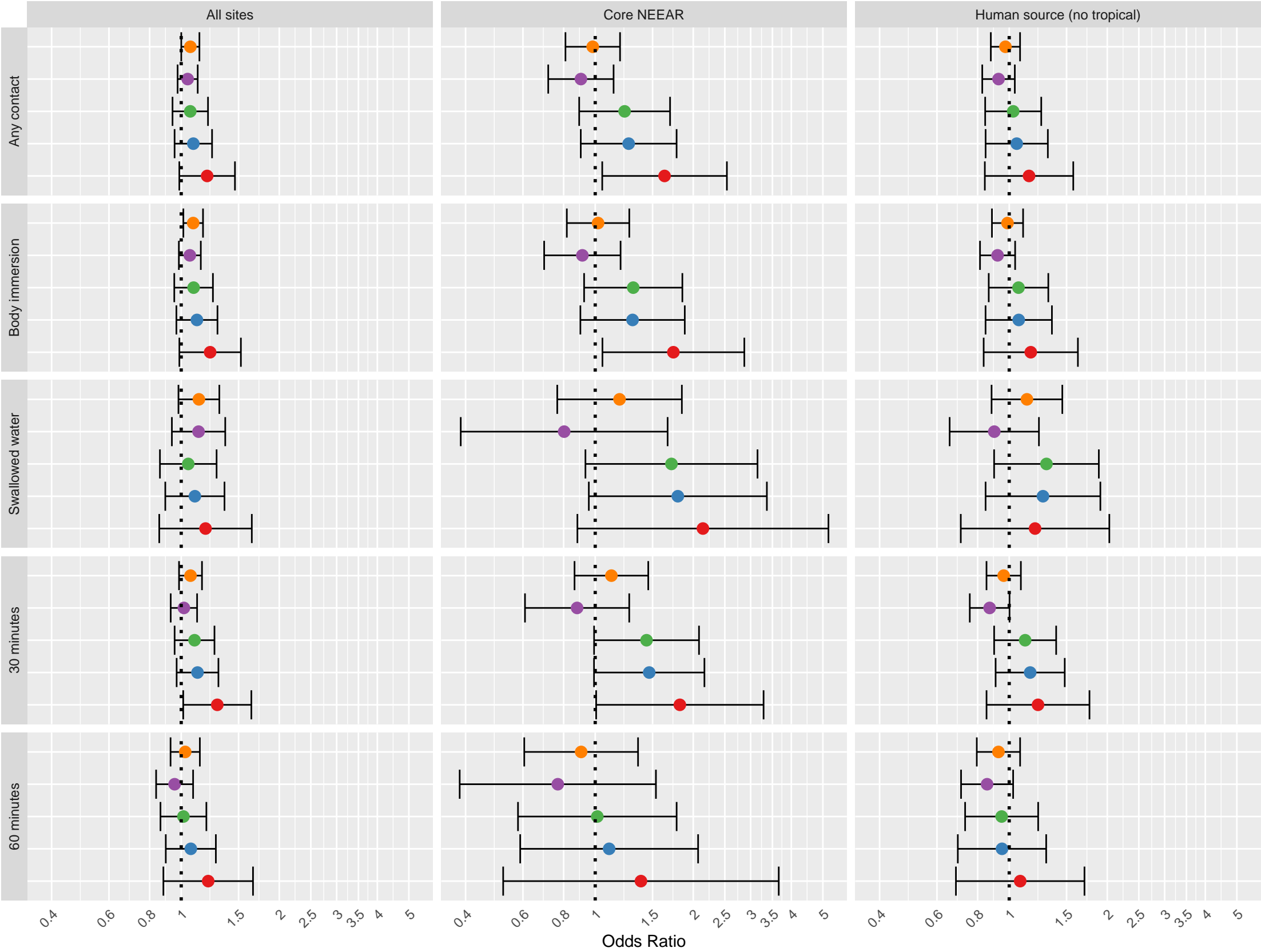

Supplement: S11 Fig — (PDF) [file pone.0266749.s015.pdf]

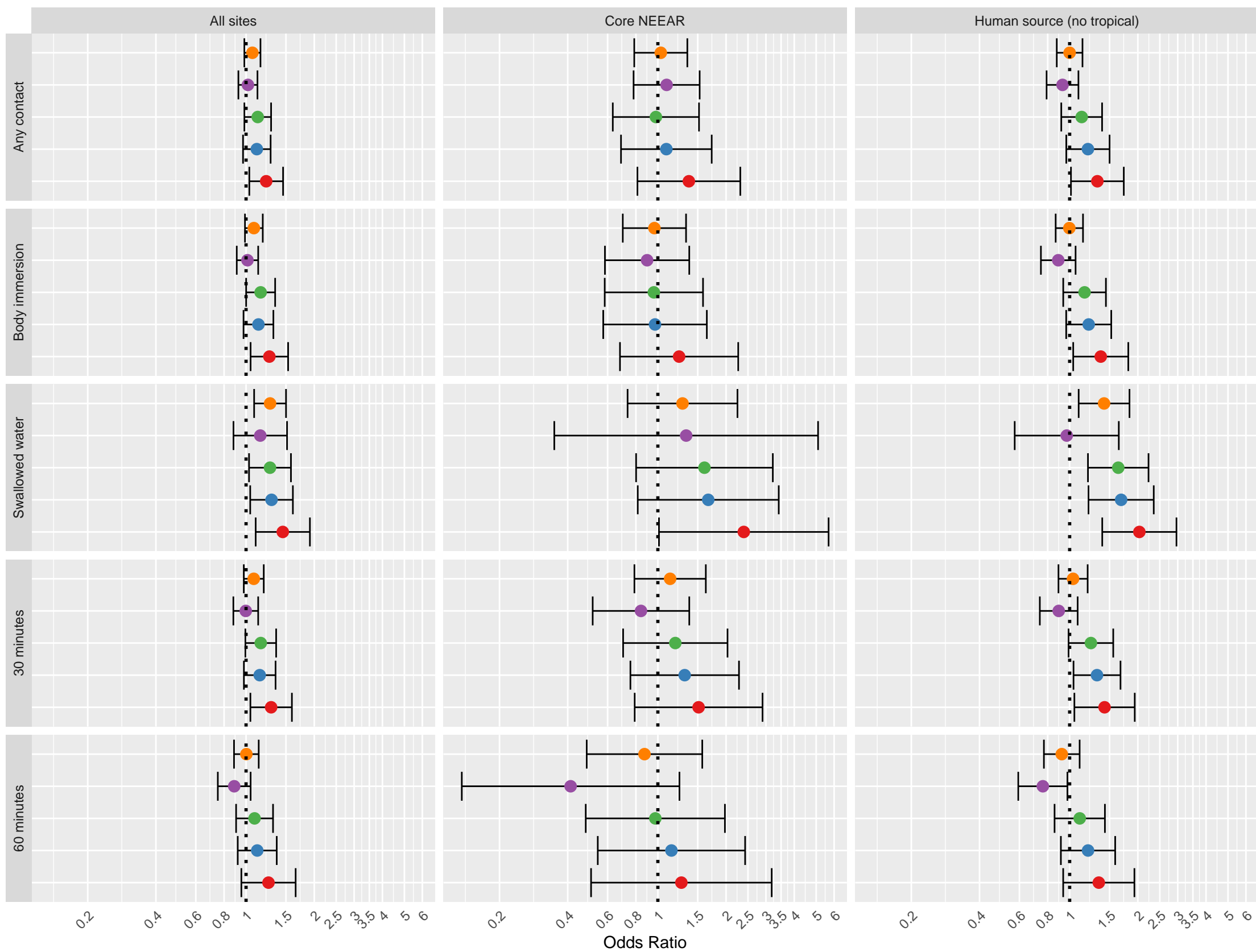

Supplement: S12 Fig — (PDF) [file pone.0266749.s016.pdf]

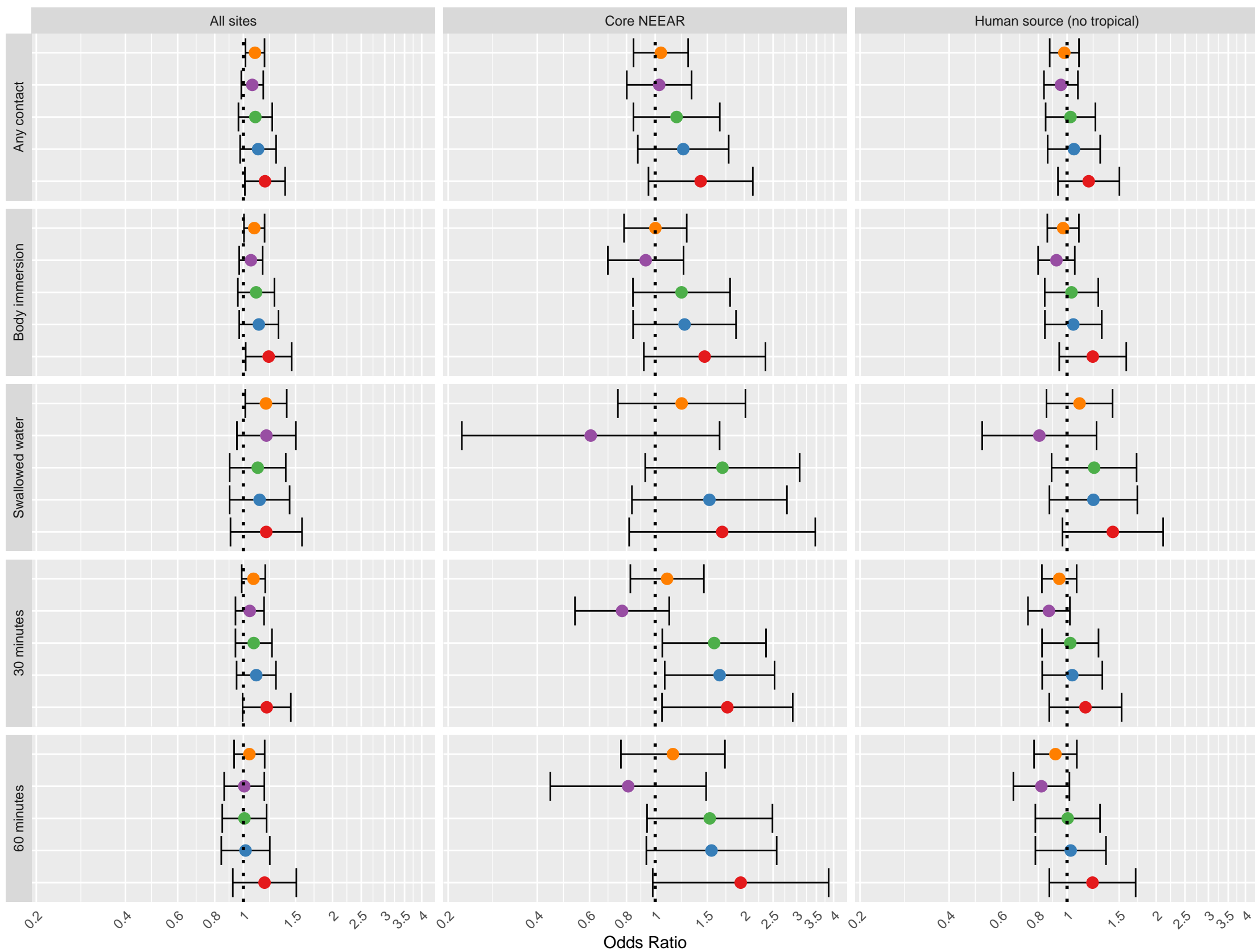

Supplement: S13 Fig — (PDF) [file pone.0266749.s017.pdf]

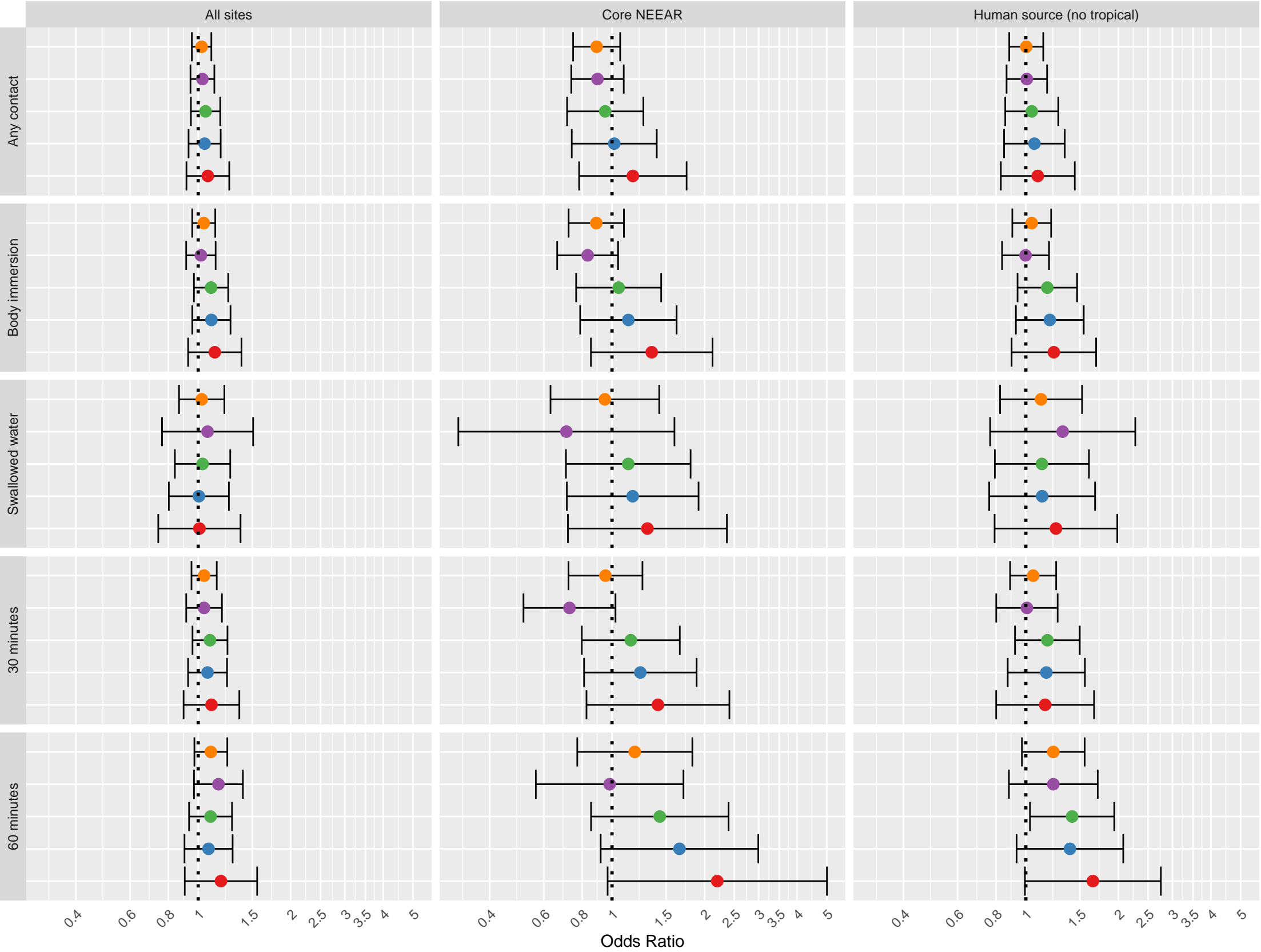

Supplement: S14 Fig — (PDF) [file pone.0266749.s018.pdf]

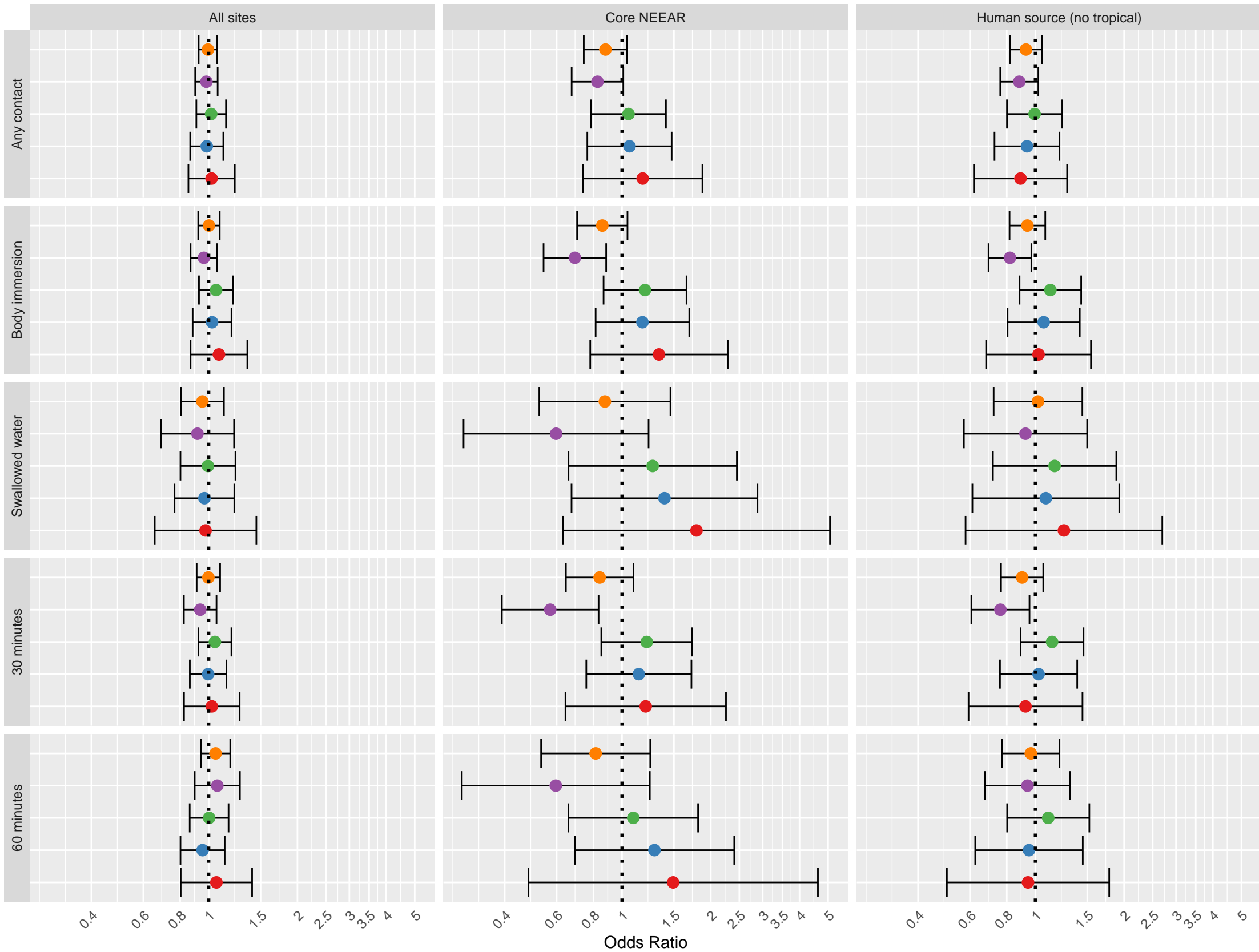

Supplement: S15 Fig — (PDF) [file pone.0266749.s019.pdf]

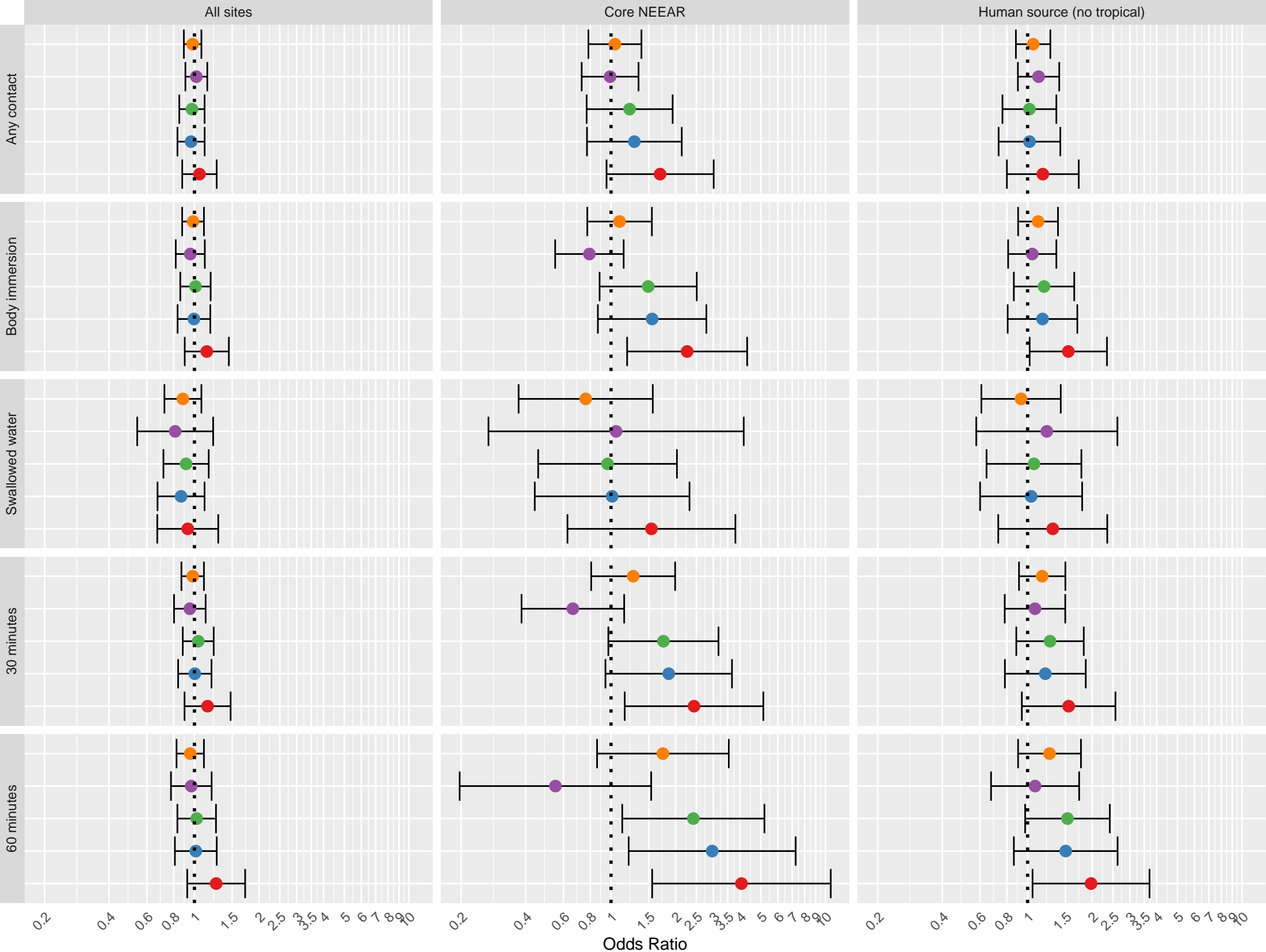

Supplement: S16 Fig — (PDF) [file pone.0266749.s020.pdf]

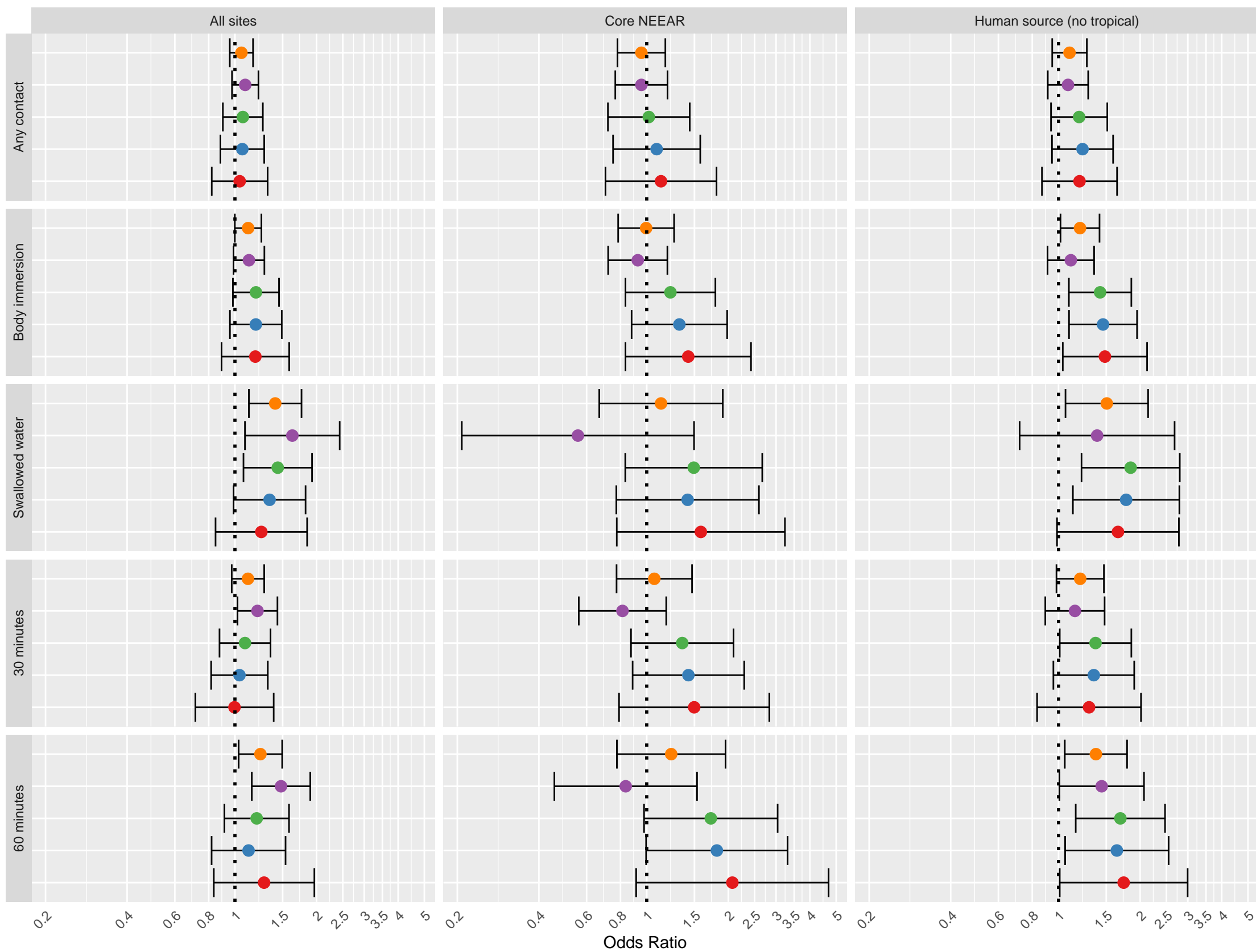

Supplement: S17 Fig — (PDF) [file pone.0266749.s021.pdf]

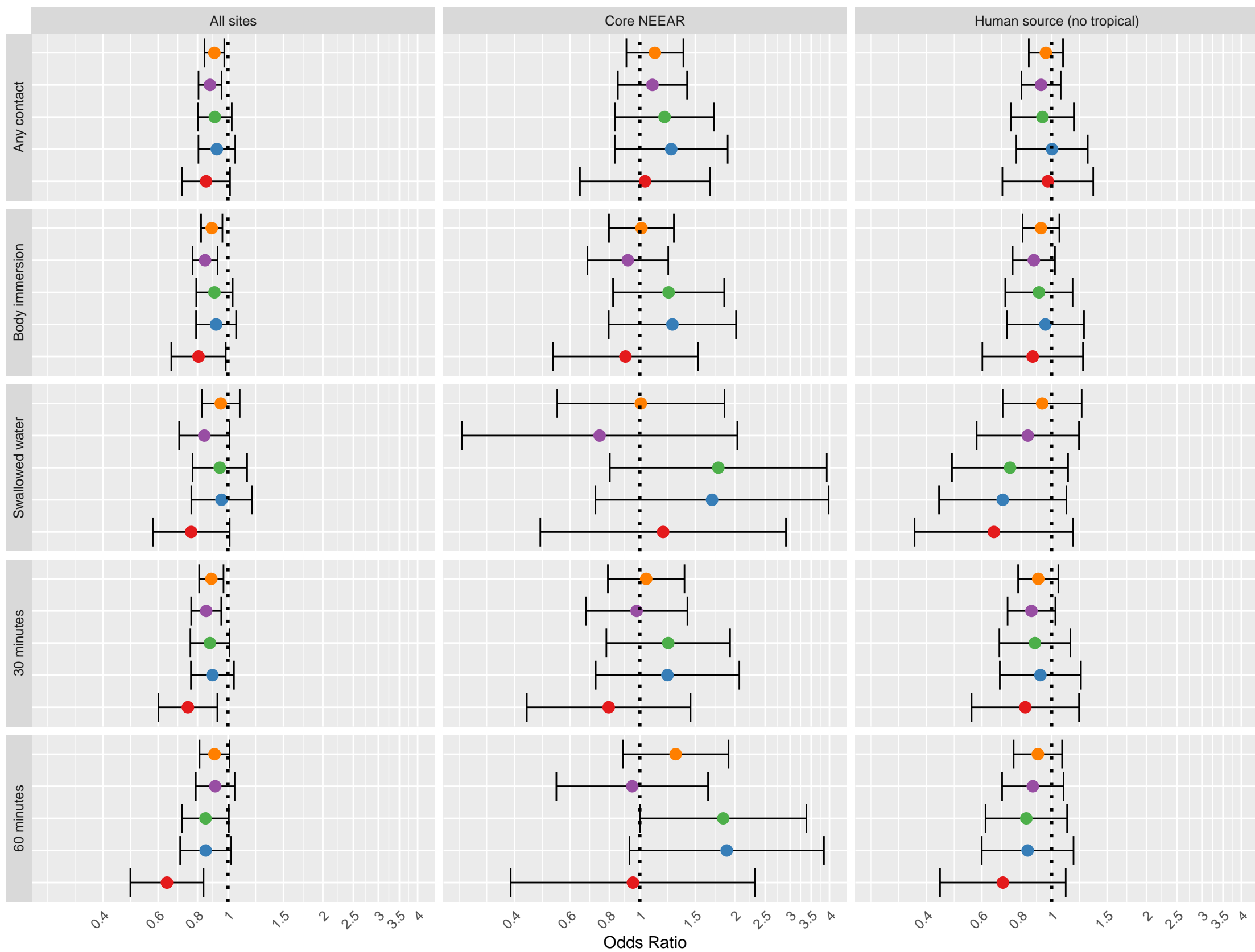

Supplement: S18 Fig — (PDF) [file pone.0266749.s022.pdf]

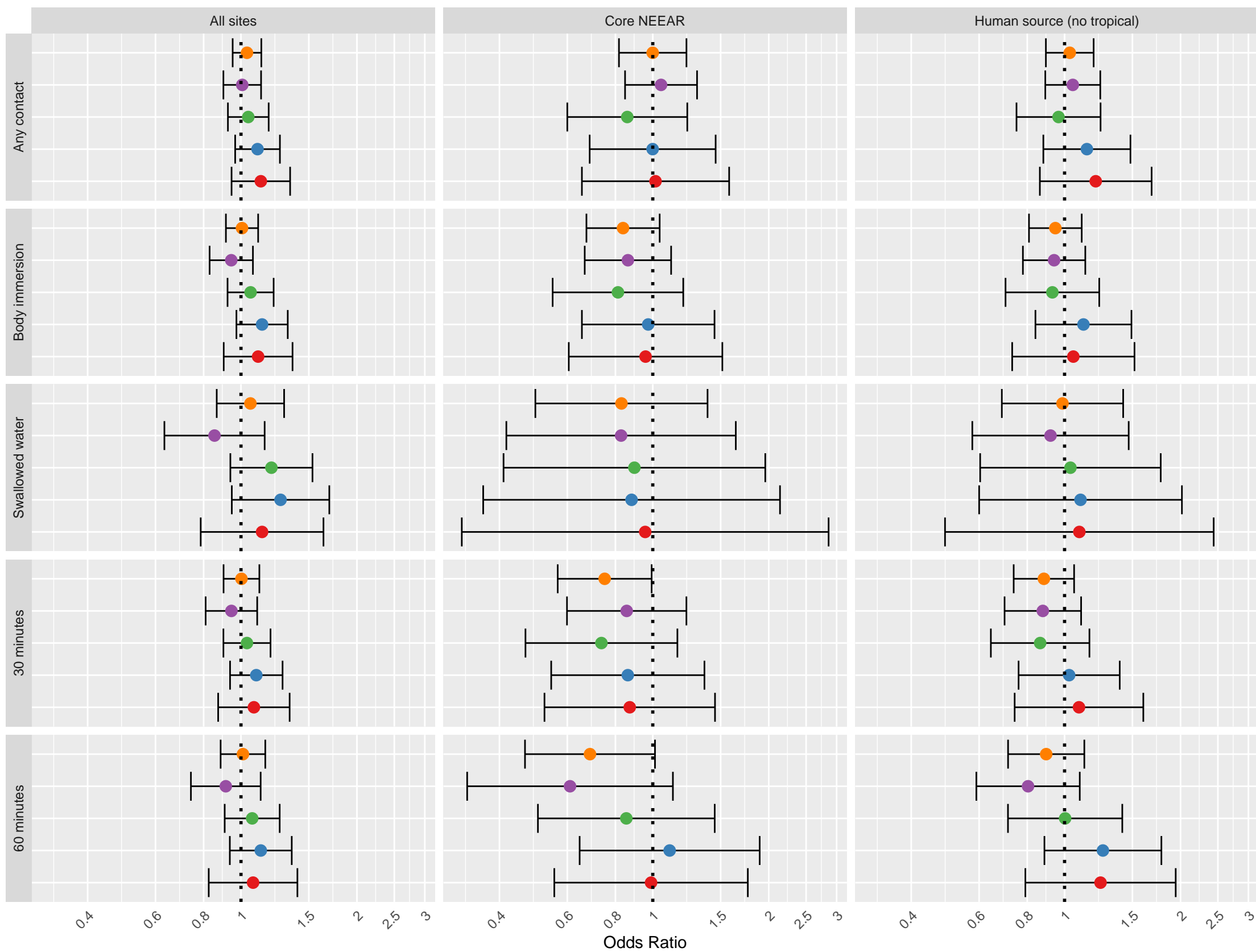

Supplement: S19 Fig — (PDF) [file pone.0266749.s023.pdf]

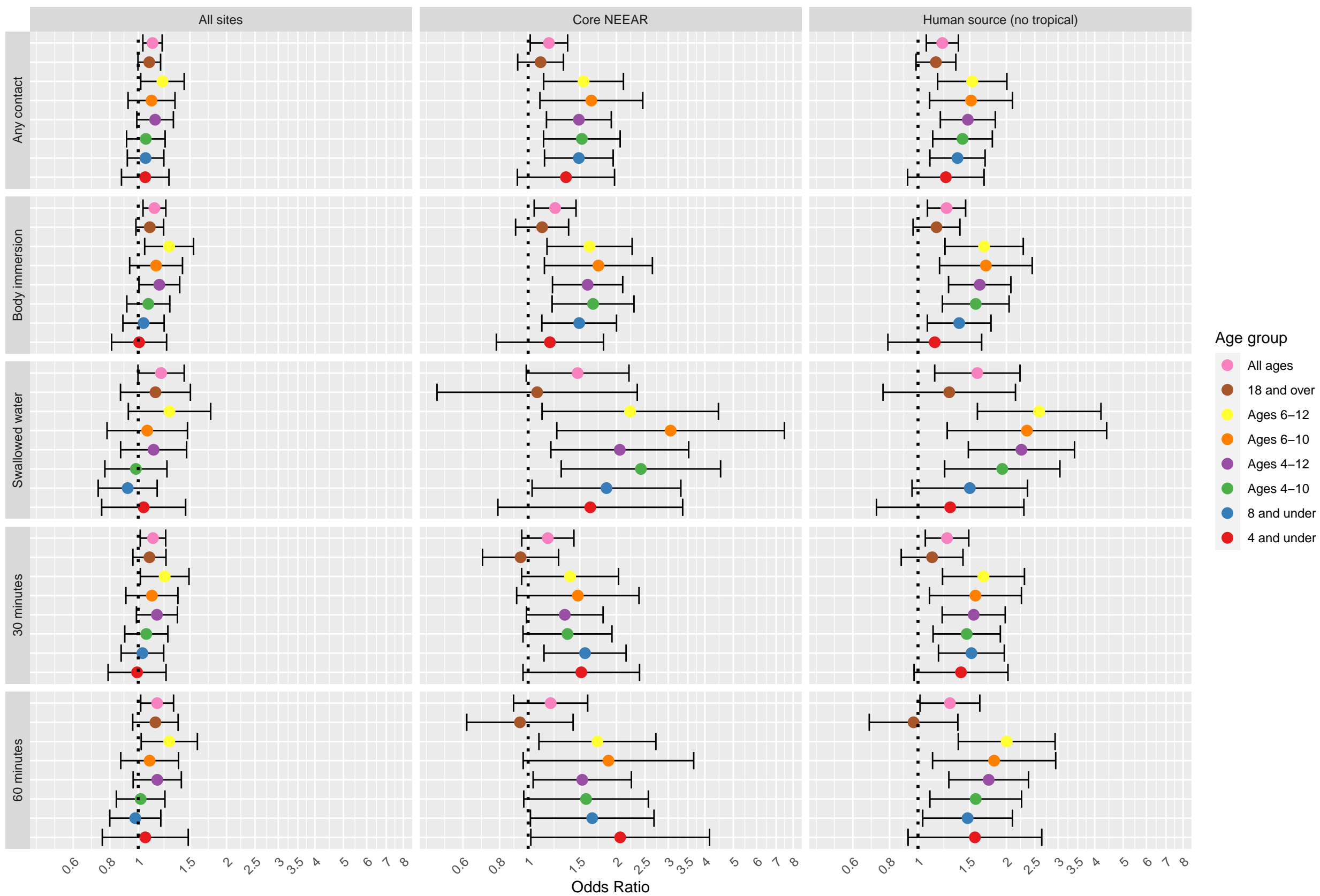

Supplement: S20 Fig — (PDF) [file pone.0266749.s024.pdf]
